# Supplementary material for: Open Humans: A platform for participant-centered research and personal data exploration
Source: Gigascience. 2019 Jun 26;8(6):giz076. doi: 10.1093/gigascience/giz076 (PMC6593360; doi:10.1093/gigascience/giz076)
Supplement: giz076_GIGA-D-18-00451_Original_Submission [file giz076_giga-d-18-00451_original_submission.pdf]

GigaScience

Open Humans: A platform for participant-centered research and personal data exploration

--Manuscript Draft--

**Manuscript Number:**	GIGA-D-18-00451
**Full Title:**	Open Humans: A platform for participant-centered research and personal data exploration
**Article Type:**	Review
**Funding Information:**	Robert Wood Johnson Foundation (NA) — Not applicable<br>John S. and James L. Knight Foundation (NA) — Not applicable
**Abstract:**	Background: Many aspects of our lives are now digitized and connected to the internet. As a result, individuals are now creating and collecting more personal data than ever before. This offers an unprecedented chance for fields of human subject research ranging from the social sciences to precision medicine. With this potential wealth of data come practical problems - such as how to merge data streams from various sources - as well as ethical problems - how can people responsibly share their personal information?<br>Results: To address these problems we present Open Humans, a community-based platform that enables personal data collections across data streams, enables individuals to take control of their personal data, and enables academic research as well as patient-led projects. We showcase data streams that Open Humans combines - such as personal genetic data, wearable activity monitors, GPS location records and continuous glucose monitor data - along with use cases of how that data is used by various participants.<br>Conclusions:<br>Open Humans highlights how a community-centric ecosystem can be used to aggregate personal data from various sources as well as how these data can be ethically used by academic and citizen scientists.
**Corresponding Author:**	Bastian Greshake Tzovaras, Ph.D<br>E O Lawrence Berkeley National Laboratory<br>UNITED STATES
**Corresponding Author Secondary Information:**	
**Corresponding Author's Institution:**	E O Lawrence Berkeley National Laboratory
**Corresponding Author's Secondary Institution:**	
**First Author:**	Kevin Arvai
**First Author Secondary Information:**	
**Order of Authors:**	Kevin Arvai
	Mairi Dulaney
	Vero Estrada-Galinanes
	Beau Gunderson
	Tim Head
	Dana Lewis
	Oded Nov
	Orit Shaer
	Jason Bobe

Powered by Editorial Manager® and ProduXion Manager® from Aries Systems Corporation

	Mad Price Ball
	Bastian Greshake Tzovaras, Ph.D
**Order of Authors Secondary Information:**	
**Additional Information:**	
**Question**	**Response**
Are you submitting this manuscript to a special series or article collection?	No
**Experimental design and statistics**<br><br>Full details of the experimental design and statistical methods used should be given in the Methods section, as detailed in our Minimum Standards Reporting Checklist. Information essential to interpreting the data presented should be made available in the figure legends.<br><br>Have you included all the information requested in your manuscript?	Yes
**Resources**<br><br>A description of all resources used, including antibodies, cell lines, animals and software tools, with enough information to allow them to be uniquely identified, should be included in the Methods section. Authors are strongly encouraged to cite Research Resource Identifiers (RRIDs) for antibodies, model organisms and tools, where possible.<br><br>Have you included the information requested as detailed in our Minimum Standards Reporting Checklist?	Yes
**Availability of data and materials**<br><br>All datasets and code on which the conclusions of the paper rely must be either included in your submission or deposited in publicly available repositories (where available and ethically appropriate), referencing such data using	Yes

a unique identifier in the references and in the “Availability of Data and Materials” section of your manuscript.<br><br>Have you have met the above requirement as detailed in our Minimum Standards Reporting Checklist?	

Powered by Editorial Manager® and ProduXion Manager® from Aries Systems Corporation

Manuscript
Click here to access/download;Manuscript;msfile-1.pdf
Click here to view linked References

GigaScience, 2017, 1–11
doi: xx.xxxx/xxxx
Manuscript in Preparation
Paper

PAPER

Open Humans: A platform for participant-centered research and personal data exploration

Bastian Greshake Tzovaras[1,2,*], Kevin Arvai , Mairi Dulaney[1], Vero Estrada-Galiñanes[3], Beau Gunderson , Tim Head[4], Dana Lewis[5], Oded Nov[6], Orit Shaer[7], Jason Bobe[8] and Mad Price Ball[1,*]

[1]Open Humans Foundation, USA and [2]Lawrence Berkeley National Laboratory, Berkeley, CA, USA and [3]QoL Lab, Department of Computer Science, University of Copenhagen, Denmark and [4]Wild Tree Tech, Switzerland and [5]OpenAPS, Seattle, WA, USA and [6]Tandon School of Engineering, New York University, New York, USA and [7]Wellesley College, Wellesley, MA, USA and [8]Institute for Next Generation Healthcare, Icahn School of Medicine at Mount Sinai, NY, USA

[*]bgreshake@gmail.com; mpball@gmail.com

Abstract

Background: Many aspects of our lives are now digitized and connected to the internet. As a result, individuals are now creating and collecting more personal data than ever before. This offers an unprecedented chance for fields of human subject research ranging from the social sciences to precision medicine. With this potential wealth of data come practical problems – such as how to merge data streams from various sources – as well as ethical problems – how can people responsibly share their personal information? **Results:** To address these problems we present Open Humans, a community-based platform that enables personal data collections across data streams, enables individuals to take control of their personal data, and enables academic research as well as patient-led projects. We showcase data streams that Open Humans combines – such as personal genetic data, wearable activity monitors, GPS location records and continuous glucose monitor data – along with use cases of how that data is used by various participants. **Conclusions:** Open Humans highlights how a community-centric ecosystem can be used to aggregate personal data from various sources as well as how these data can be ethically used by academic and citizen scientists.

Key words: Personal Data; Crowdsourcing; Citizen Science; Database; Open Data ; Participatory Science; Peer Production

Background

Human subject research at large, from biomedical & health research to the social sciences, is experiencing rapid changes. The rise of electronic records, online platforms, and data from devices contribute to a sense that these collected data can change how research in these fields is performed [1, 2, 3, 4]

Among the impacted disciplines is precision medicine – which takes behavioral, environmental, and genetic factors into account and has become a vision for health-care in the United States [5]. By taking individual parameters into account, precision medicine aims to improve health outcomes, for example by optimizing drugs based on a patient's genetic makeup [6, 7].

Access to large-scale data sets, along with an availability of appropriate methods to analyze these data [8, 9], is often described as a major prerequisite for the success of precision medicine [10]. Dropping costs for large-scale, individualized analyses such as whole-genome sequencing [11] help facilitate both research of precision medicine and its adoption. In addition, an increasing number of patients and healthy individuals are collecting health-related data outside traditional health-

Compiled on: November 12, 2018.
Draft manuscript prepared by the author.

care, for example through smartphones and wearable devices [12, 13] or through direct-to-consumer (DTC) genetic testing [14].

Indeed, an estimated 12-17 million individuals have taken a DTC genetic test [15, 16] and it is estimated that by 2020 over 2 exabytes of storage will be needed for health care data [17] alone. Furthermore, data from social network sites like Facebook or Twitter are becoming more and more interesting for medical data mining [18]. Additionally, more data is becoming available from personal medical devices, both in real-time and for retrospective analysis [19].

These changes to research and medical practice bring with them a number of challenges that need to be solved, including the problems of data silos, ethical data sharing and participant involvement.

Data Silos

To fully realize the promises of these large personal data collections, not only in precision medicine but all fields of research, access to both big data and smaller data sources is needed, as well as the ability to tap into a variety of data streams and link these data [20, 10]. Data silos can hinder the merging of data for a number of reasons: Data silos can be incompatible due to different data licenses [21] or inaccessible due to privacy and ethical concerns [22, 23, 24].

Furthermore, in the case of wearable devices, social media and other data held by companies, data exports are often not available. In other cases data access is legally mandated, but the practical outcomes are mixed [25] or in progress, *e.g.* for clinical health data in the United States as mandated by the 1996 *Health Insurance Portability and Accountability Act* and 2009 *Health Information Technology for Economic and Clinical Health Act* (HIPAA and HITECH Act), and for personal data in the European Union as mandated by the 2016 *General Data Protection Regulation* (GDPR) [26, 27]. In addition, within the context of human subjects research, data access may be recommended [28] but not legally required, and as a result is not typically provided [29]. Data portability by individuals has potential value for research, as an individual's ability to access, manage, and transfer copies of their data empowers them to be a key data holder for precision medicine frameworks.

Ethical Data Re-Use

While the sharing and re-using of biomedical data can potentially transform medical care and medical research, it brings along a number of ethical considerations [30, 31]. In the field of human genetics, the ethics of sharing data has been extensively evaluated with respect to how research participants and patients can give informed consent with respects to genetic discrimination, loss of privacy, and the risks of re-identification in publicly shared data [32, 33]. Due to access and portability issues, however, research with biomedical data is rarely driven by the individuals data came from – and as a result, fails to give patients much power over how their data can be used [34].

Social media is also gaining importance in research as well as public health [35]. Differing perceptions on the sensitivity of social media data can lead to privacy concerns, e.g. as occurred with an analysis performed on 70,000 users of an online dating website, where private personal data was scraped by researchers and then publicly shared[36]. Such cases have sparked calls for caution in performing "big data" research with these new forms of personal data [37, 38].

Research which interacts with social media users raises additional concerns. For example, Facebook was widely criticized for an experiment to study emotional contagion on 700,000 of its users without their consent or debriefing, prompting discussion of the ethics of unregulated human subjects research and "A/B testing" by private entities [39, 40, 41]. At the same time, the Cambridge Analytica controversy has led Facebook to tighten control over their API, turning it even more into a silo that does not allow for research to be done by outside researchers [42].

For the foreseeable future, research that re-uses data from commercial interests will have to decide how to balance the interests of commercial data sources, data subjects, and the larger good to society. While there is no consensus on how research consent for existing personal data should be performed, participants have a wish to consent and control their data [43]. Putting participants into control of their data will be more central in the more sensitive context of precision medicine [23].

Participant Involvement

Citizen science mostly describes the involvement of volunteers in the data collection, analysis, and interpretation phases of research projects [44], thus both supporting the research process itself and helping with public engagement. Along with these reasons to actively involve volunteers, there is a case to be made to see participatory science included in the Humans Right for Science [45].

Traditionally, many participatory science projects focused on the natural sciences, like natural resource management, environmental monitoring/protection, and astrophysics [46, 47, 48]. In many of these examples volunteers are asked to crowdsource and support scientists in the collection of data - e.g. by field observations or through sensors [49] or to perform human computation tasks, e.g. to classify images [50] or to generate protein-structure foldings [51].

Analogous to the movement in other fields, there is a growing movement for more participant/patient involvement in human subject research, including fields such as radiology, public health, psychology, and epidemiology [52, 53]. It furthermore has been recognized that patients often have a better understanding of their disease and needs than medical/research professionals [54, 55] and that patient involvement can help catalyze policy interventions [56]. Examples include the studies on amyotrophic lateral sclerosis initiated by *PatientsLikeMe* users [57], crowd-sourcing efforts like *American Gut* [58], and a variety of other *citizen genomics* efforts [59]. It is estimated that involving patients in clinical research can not only help in minimizing cost but more importantly also lead to drugs being brought to market much earlier than otherwise [60].

The *Quantified Self* movement, in which individuals perform self-tracking of biological, behavioral, or environmental information and design experiments with an *n=1* to learn about themselves [61], can be seen in this continuum of participant-led research [62]. By performing self-experiments and recording their own data, individuals are gaining critical knowledge about themselves and the process of performing research.

A participant-centered approach to research

As shown above, substantially involving patients and participants in the research process has multiple benefits. Participants as primary data holders can help in breaking down walls between data silos to aggregate and share their personal data streams. Furthermore, by being involved in the research process and actively providing data, they gain autonomy and can actively consent to their data being used - thus reducing ethical concerns. Last, but not least, active research participants can give valuable input from their perspectives, leading to better research.

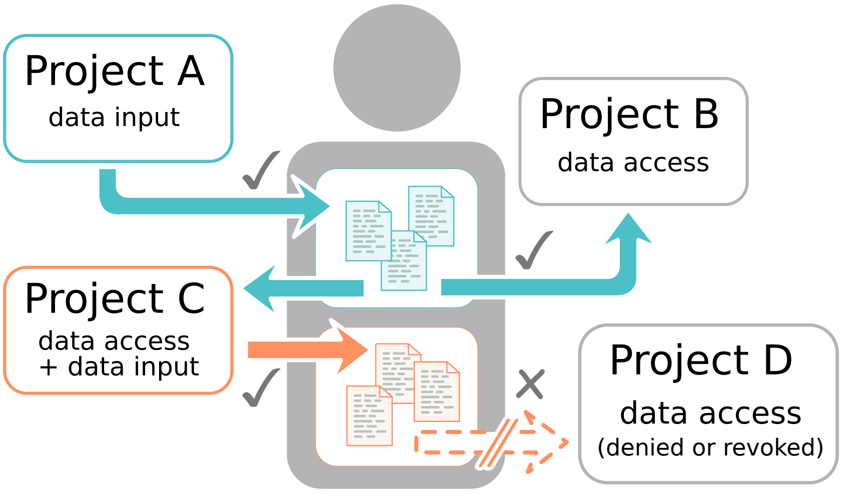

Figure 1. The Open Humans authorization flow. A *Member* (center) can join *Projects* and approve them to read or write *Data*. The *Member* approves *Project A* to deposit files (blue) into their account. They also approve *Project B* to read the files that *Project A* has deposited. Additionally, the *Member* approves *Project C* to both read the files of *Project A* and write new files. The *Member* declines to give access to their personal data to *Project D*.

In recent years a number of projects have started to explore both data donations and crowd-sourcing research with an extended involvement of participants. In the fields of genomics, both academic projects like *DNA.Land* [63] and community-driven projects like *openSNP* [64] are enabling crowdsourcing via personal genetic data set donations. Furthermore, the idea of *Health Data Cooperatives* that are communally run to manage access to health data has emerged [23].

However, most of these projects limit participants' involvement in the research process: a participant is limited to providing data for a data repository. Furthermore, most of these projects are not designed to effectively bundle different data streams, but focus on a specific kind of data. Additionally, participants are rarely given an easy way to help in designing a study or even running their own.

To close these gaps we developed *Open Humans*, a community-based platform that enables its members to share a growing number of personal data types; participate in research projects and create their own; and facilitates the exploration of personal data for the individual member. *Open Humans* was initially conceived as an iteration on work with the Harvard Personal Genome Project [65]. Along with the platform itself, we present a set of examples on how the platform is already used for academic and participant-led research projects.

Results

We designed *Open Humans* as a web platform with the goal of easily enabling connections to existing and newly created data sources and data (re-)using applications. The goal of the platform is to enable members to import data into their accounts from various sources and use the data to explore it on their own and share it with citizen science and academic research projects alike.

Design

In the center of the design are three main components: *Members*, *Projects* and *Data* objects. *Members* can join various *Projects* and authorize them to read *Data* that's stored in their account as well as write new *Data* for this *Member* (see Figure 1 for a dataflow diagram).

Projects

Projects are the primary way for *Members* to interact with *Open Humans*. As *Projects* can be created by any member, they are not limited to academic research projects but open to participant-led projects, too. During project creation a prospective project lead will not only give a description of their project, but also specify the access permissions they request from members that decide to join. These permissions may include:

Username By default projects do not get access to a members username; each member is identified with a random, unique identifier specific to that project. This way members can join a project while being pseudonymous.

Data Access A *Project* may ask permission to read *Data* that have been deposited into a member's account by other projects. A project lead needs to specify to which existing projects' data they want to have access to and only this data will be shared with the new project.

Through the permission system, members get a clear idea of the amount of *Data* they are sharing by joining a given *Project* and whether their username will be shared. Furthermore, new *Data* can be deposited into the accounts of *Members* that have joined a project. Through this, projects are also the method through which data is added to *Member* accounts. In addition to specifying the access permissions, projects also need to clearly signal whether they are a research study that has been approved by an Institutional Review Board (IRB) or equivalent, or whether they are a project not performing such research (i.e. not subject to this oversight).

Projects can be set up in two different ways: As an *on-site project* or as an *OAuth2 project*. While an on-site format minimizes the need for technical integrations on the side of the project, access to the *Data* shared with it can not easily be automated and requires manual interactions.

OAuth2 projects on the other hand require a larger effort to implement the *OAuth2* authentication methods. In return they offer ongoing programmatic access to the shared data, making it well-suited for connecting to other web or smartphone applications.

Given this very broad classification, a *Project* can cover anything from data import projects, to research projects, to self-

quantification tools which visualize and analyze a member's data.

Members

Members interact with *Projects* that are run on *Open Humans*. By joining projects that act as data uploaders, they can add specific *Data* into their Open Humans accounts. This is a way to connect external services: e.g. put their genetic data or activity tracking data into their *Open Humans* account. Once they have connected to relevant *Projects* that import their own data, members can opt-in to joining additional *Projects* that they wish to grant access to their account's data.

As *Members* are able to selectively join *Projects*, they keep full control over how much of their *Data* files they want to share and with which *Projects*.

Data input and management

Data is uploaded into a *Member*'s account, which allows any joined *Projects* with requisite permissions to access this data. To be fully universal to all the possible projects that can be run on *Open Humans*, all data are stored in files that can be downloaded by users and *Projects* that got permission. For any file that a *Project* deposits in turn into a *Member*'s account, the uploading *Project* needs to specify at least a description and tags as meta data for the files.

Members can always review and access the *Data* stored in their own accounts. By default, the *Data* uploaded into their accounts is not shared with any projects but the one that deposited the data, unless and until other *Projects* are joined and specifically authorized to access this data. In addition to being able to share data with other *Projects*, members can also opt-in into making the data of individual projects publicly available. *Data* that has been publicly shared is then discoverable through the *Open Humans* Public Data API, and is potentially visible on a *Member*'s user profile.

Open Humans in Practice

Using this design, a number of projects that import data into *Open Humans* are provided directly by *Open Humans*. Among data sources that can be imported and connected are *23andMe*, *AncestryDNA*, *Fitbit*, *Runkeeper*, *Withings*, *uBiome* and a generic *VCF* importer for genetic data like whole exome or genome sequencing. Furthermore, as a special category, the *Data Selfie* project allows members to add additional data files that are not supported by a specialized project yet.

The community around the *Open Humans* platform has expanded the support to additional *Data* sources by writing their own data importers and data connections. These include a bridge to *openSNP*, and importers for data from *FamilyTreeDNA*, *Apple HealthKit*, *Gencove*, *Twitter* and the *Nightscout* (open source diabetes) community. Across these data importers, the platform supports data sources covering genetic and activity tracking data as well as recorded GPS tracks, data from glucose monitors, and social media.

The platform has grown significantly since its launch in 2015: As of November 12th 2018, 6,143 members have signed up with *Open Humans*. Of these, 2,457 members have loaded 16,081 data sets into their accounts. In cases where external data sources support the import of historical data (e.g. Fitbit, Twitter), data sets can include data that reaches back before the launch of Open Humans. Furthermore, overall there are 30 projects that are actively running on Open Humans, with an additional 12 projects that have already finished data collection and thus have been concluded (see Table 1 for the most used projects).

Use Cases

To demonstrate the range of projects made possible through the platform and how the community improves the ecosystem that is growing around *Open Humans* we highlight some of the existing projects, covering both participant-led as well as academic research and the self-quantification community.

OpenAPS and Nightscout Data & Data Commons

There are a variety of open source diabetes tools and applications that have been created to aid individuals with type 1 diabetes in managing and visualizing their diabetes data from disparate devices. One such tool is Nightscout. Another such example is OpenAPS, the Open Source Artificial Pancreas System, which enables individuals to utilize existing insulin pumps and continuous glucose monitors (CGMs) with off-the-shelf hardware and open source software as a hybrid closed loop "artificial pancreas" system [66]. These platforms and tools enable real-time and retrospective data analysis of rich and complex diabetes data sets from the real world.

Traditionally, gathering this level of diabetes data would be time-consuming, expensive, and otherwise burdensome to the traditional researcher, and often a full barrier to researchers interested in getting started in the area of diabetes research and development. Using Open Humans, individuals from the diabetes community have created a data uploader tool Nightscout Data Transfer Tool to enable individuals to anonymously upload their diabetes data from Nightscout and/or OpenAPS [67]. This enables an individual to protect their privacy, and also only upload data to one place while facilitating its usage in multiple studies and projects. These two data commons have simple requirements for use, allowing any traditional *or* citizen science (e.g. patient) researcher who would like to utilize this data for research. These data commons were created with the goal of facilitating more access to diabetes data such as CGM datasets that are traditionally expensive to access, enabling more researchers to explore innovations for people with diabetes. Additionally, OpenAPS is the first open source artificial pancreas system with hundreds of users; there is benefit in openly sharing the data from users, who are hoping such data sharing will facilitate better tools and better innovations for academic and commercial innovations in this space. To date, dozens of researchers and many community members have accessed and utilized data from each of these commons. Some publications and presentations have also been completed, showcasing the work and the data donated by members of the community, and further allowing other researchers to build on this body of work and these data sets [68] (https://openaps.org/outcomes/).

In addition to facilitating easier access to more and richer diabetes data, this community has also been developing a series of open source tools to enable individuals to more easily work with the datasets (https://github.com/danamlewis/OpenHumansDataTools). Many researchers are most comfortable with csv formatted data, whereas the diabetes data is uploaded as json files. Additionally, because of the plethora of devices and options of how and under what name data is uploaded, the json has an infinite range of possibilities for the structure of the schema. As a result, the open source toolset began to be developed to first enable easy conversation of the complex json into csv, and has been followed by additional tools with additional documentation to facilitate selecting data elements for further analysis out of the dataset.

Connecting an existing, open database: openSNP

openSNP is an open database for personal genomics data which allows individuals to donate the raw DTC genetic test data into the public domain [64]. So far, over 4,500 genetic data sets have been donated to *openSNP*, making it one of the largest

Table 1. *Open Humans* projects with more than 200 members

Project name	Description	Members	Data deposited	Data access requested
23andMe Upload	Enables members to import their 23andMe data	1054	23andMe data	-
Harvard Personal Genome Project	Enables members to import their data from the Personal Genome Project	816	Full genome sequencing data & survey data	-
Genevieve Genome Report	Matches a member's genome against public variant data, and invites them to contribute to shared notes.	749	-	23andMe Upload, Harvard PGP, Genome/Exome Upload, Username & public data
Keeping Pace	Seeks to study data about how we move around, to understand how seasons and local environment influence our movement patterns.	390	-	Fitbit, Jawbone, Moves, Apple HealthKit, Runkeeper
AncestryDNA Upload	Enables members to import their AncestryDNA data	378	AncestyDNA data	-
Fitbit Connection	Connect a member's Fitbit account to add data from their Fitbit activity trackers and other Fitbit devices.	368	Data from a Fitbit account	-
Personal Data Notebooks	Enables personal data analyses with Jupyter Notebooks	361	Jupyter Notebooks	-
GenomiX Genome Exploration	A study of how people interact with their genome data using GenomiX, a visualization tool	326	-	Username & public data
Twitter Archive Analyzer	Enables members to import their Twitter archives and analyzes them	305	Twitter archives	-
Circles	A research study that aims to discover the genetic basis for a mysterious and remarkable human trait: the areola.	303	-	23andMe, AncestryDNA, Data Selfies, Harvard PGP, Genome/Exome Upload
openSNP	Enables members to connect their *Open Humans* and *openSNP* accounts	255	openSNP user details	Username & public data
Nightscout Data Transfer	A tool to easily enable the upload of data from individual Nightscout databases	246	Nightscout data	-
Runkeeper	Imports a member's data from Runkeeper	210	Runkeeper data	-

Data was collected on 2018-11-12

crowdsourced genome databases. While people can annotate their genomes with additional phenotypes on *openSNP*, there is no integration of further data sources into *openSNP*. To further enrich a member's account on both *Open Humans* and *openSNP*, a project that connects the two was started.

The *openSNP* project for *Open Humans* asks members for permission to read their *Open Humans* username during the authentication phase. By publicly recording a members *Open Humans* username, it is then possible to link the public data sets on *Open Humans* to a given *openSNP member*. Additionally, *openSNP* also deposits a link to a member's public *openSNP* data sets in their *Open Humans* member account. Through this other *Open Humans* projects can ask individuals to get access to their genetic data and phenotypes stored on *openSNP*. So far over 250 people have taken advantage of linking their *openSNP* and *Open Humans* accounts.

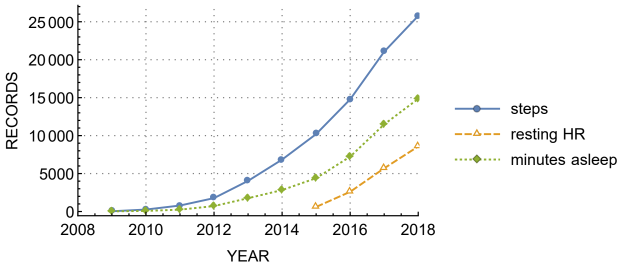

Figure 2. Self-quantification data from *Fitbit* project. Number of public records from January 2009 until October 2018 (cumulative total).

Genetic Data Augmentation

Most DTC genetic testing companies genotype customers using single-nucleotide polymorphism (SNP) genotyping technology which genotypes a fraction of the total available sites in a human genome. As any two human genomes are more than 99% identical, these genotyped sites are carefully selected to capture human variation across global sub-populations. These sites (or genetic *variants*) can inform customers about their genetic ancestry, predict traits such as eye color, and even determine susceptibility to some recessive diseases. While DTC testing may only genotype a fraction of total sites available in the genome, it's offered at a fraction of the price when compared to more comprehensive genotyping methods such as exome or genome sequencing. Until recently, individuals who wanted to know their genotypes at sites not covered by DTC testing needed to purchase a significantly more expensive, albeit comprehensive genotyping test.

Genome-wide genotype imputation is an increasingly popular technique that offers a no- or low-cost alternative to comprehensive genotyping methods. In short, imputation is performed by scanning the entire genome in large intervals and using high-quality genotype calls from a large reference population to statistically determine a sample's (or samples') genotype likelihoods at missing sites based on shared genotypes with the reference population. Traditionally, genotype imputation has not been readily accessible to DTC customers because it entails a complex multi-step process requiring technical expertise and computing resources. Recently, the Michigan Imputation Server launched a free to use imputation pipeline [69]. The server was designed to be user-friendly and greatly lowered the barrier to entry for everyday DTC customers to have access to imputed genotypes.

Imputer is a participant-created project that performs genome-wide genotype imputation on one of a *member's* connected genetic data sources, such as *23andMe* or *AncestryDNA*. Once connected via *OAuth2*, the *Imputer* interface (http://openimpute.com) allows *members* to select which genetic data source they would like to impute and launches the imputation pipeline in one click. *Imputer* submits the imputation job to a queue on a server where the imputation is performed. Once the job has finished, the imputed genotypes are uploaded as a *.vcf* file and an email is sent to the *member* notifying them that their data is available. *Imputer* makes it easy for *members* to augment their existing genetic data sources using techniques that have been previously difficult to access. The *Imputer* imputation pipeline was built using genipe [70] and uses the 1000 Genomes Project [71] genotype data as the reference population.

Re-use of Public Data for Understanding Health Behavior

The Quality of Life (QoL) Technologies Lab aims at improving the quality of life of individuals throughout their lives. It collects data from multiple sources to understand better the health implications of lifestyle behaviors. The goal is to leverage self-quantification data to enhance the well-being of individuals and possibly, in the long-term, reduce the prevalence of chronic diseases.

Physical inactivity is one of the strongest risk factors in preventable chronic conditions [72]. The QoL Lab assesses user's lifestyle behaviour by classifying their physical activity into different categories. For example, a member who is highly-active generates at least 12500 steps per day. Further categories allow understanding the behaviour patterns with a fine granularity. At this stage, the QoL Lab has used the *Open Humans* public dataset of *Fitbit* and *Apple HealthKit* projects. Individuals who donate public data to Fitbit and Apple HealthKit projects share with others the daily summaries taken with their *Fitbit* and *Apple* devices such steps, resting heart rate (HR) and minutes asleep. The number of records for each variable available in *Open Humans* database varies since not all the devices record the same variables and participants may choose not sharing a particular measurement, see Fig. 2.

The public datasets contain time series data from at least 30 members, who decide whether to provide access to the aforementioned measurements. The possibility of accessing public data is helpful to speed up the research done at the QoL Technologies Lab. Public data is being used to prepare algorithms that later can be applied to larger datasets, e.g. the private data. Accessing the private data as part of a research institution takes more time as it requires the approval from an Institutional Review Board which can be a lengthy process. Although public datasets are usually smaller in terms of the number of members who donate data, they are very useful for running observational studies over long periods of time. Some of the members have been tracking their activity for more than one year. *Open Humans* public donators, taken as a whole, achieved 211'861'324 steps. The earliest record dates back to January 2009, and since then, members keep donating data. Such continuity is highly valuable for researchers.

Data re-use in genetic data visualization research

With the increasing amount of individuals engaging with their genetic data, including via direct-to-consumer products, there is a need for research into how individuals interact with this data to explore and understand it. The *Human-Computer Interaction for Personal Genomics* (PGHCI) project at Wellesley College and New York University has focused on exploring these questions. Research was initially conducted by creating visualizations based on public genetic data sets, and recruiting participants via Amazon Mechanical Turk to engage with these. These data, however, were not based on a participant's *own* data, which is preferred to improve experimental validity.

Open Humans provided an opportunity to work with individuals and their data in manner that leveraged pre-existing genetic data for re-use in new research while minimizing privacy risks. A project, *GenomiX Genome Exploration*, was created in Open Humans that invited members who had publicly shared their genetic data in Open Humans to engage with a custom visualization derived from their public data. The study found various design implications in genome data engagement, including the value of affording users the flexibility to examine the same report using multiple views [73].

Personal Data Exploration

Open Humans aggregates data from multiple sources for individual members. This makes it a natural starting point for a member to explore their personal data. To facilitate this, *Open Humans* includes the *Personal Data Notebooks* project.

Through a *JupyterHub* setup (`https://jupyterhub.readthedocs.io`) that authenticates members through their *Open Humans* accounts, members can write *Jupyter Notebooks* [74] that get full access to their personal data in their web

browser. This allows members to explore and analyze their own data without the need to download or install specialized analysis software on their own computers. Furthermore, it allows members to easily analyze data across the various data sources, allowing them to find correlations.

As the notebooks themselves do not store any of the personal data, but rather the generic methods to access the data, they can be easily shared between *Open Humans* members without leaking a member's personal data. This property facilitates not only the sharing of analysis methods, but also reproducible *n=1* experiments in the spirit of self-quantification.

To make these notebooks not only interoperable and reusable, but also findable and accessible [75], the sister project to the *Personal Data Notebooks* - the *Personal Data Exploratory* - was started. Members can upload notebooks right from their *Jupyter* instance to *Open Humans* and can publish them on the *Personal Data Exploratory* with just a few clicks. The *Exploratory* publicly displays the published notebooks to the wider community and categorizes them according to the data sources used, tags and its content.

The categorization allows other members to easily discover notebooks of interest. Notebooks written by other members can be launched and run on a member's own personal data through the *Personal Data Notebooks*, requiring only a single click of a button. This close interplay between the *Personal Data Notebook* project and the *Personal Data Exploratory* project thus offers a fully integrated personal data analysis environment in which personal data can be disseminated in a secure way, while growing a library of publicly available data analysis tools.

Discussion

Participatory/Community science (also known as Citizen science) is a growing field that engages more and more people in the scientific process. But while participatory science keeps growing quickly in the environmental sciences and astronomy, its development in the humanities, social sciences, and medical research lags behind [76], despite promises for those fields [53, 77]. Both barriers in accessing personal data that is stored in commercial entities as well as legitimate ethical concerns that surround the use of personal data contribute to this slower adoption [31, 33]. *Open Humans* was designed to address many of these issues.

Granular Consent

One often suggested way to solve or minimize the ethical concerns around the sharing of personal data in a research framework is having granular privacy controls and granular consent [34]. In a medical context, most patients prefer to have a granular control over which medical data to share and for which purposes [78, 79], especially in the context of electronic medical records [80]. Furthermore, the GDPR requires data controllers to give the individual granular consent options for how their data is used [81].

Open Humans implements a granular consent and privacy model through the use of projects that members can opt-in to. On a technical level, projects need to select the data sources they would like to access, and members are shown the requested permissions during the authentication step. Additionally, projects on *Open Humans* need to adhere to the community guidelines. Among other things, these guidelines require projects to inform prospective participants about the level of data access they request, how the data will be used and what privacy & security precautions they have in place. As joining any project is optional, members retain full control over which data to share and with whom.

Data portability

Much of health data is still stored in data silos managed by national institutions, sometimes further categorized by diseases [82]. On an individual level, the situation is not much better: While medical data is stored in electronic records, much of a person's data is now held by the companies that run social media platforms, develop smartphone apps, or wearable devices [83]. This fragmentation—especially when coupled with a lack of data export methods—prevents individuals from fully making use of their own data.

Personal information management systems (PIMS) can be designed to help individuals in re-collecting and integrating their personal data from different sources [84]. The right to data portability encapsulated in the GDPR has the potential to boost the adoption of such systems, as it guarantees individuals in the European Union a right to export their personal data in electronic and other useful formats. Furthermore, both medical research [85] as well as citizen science [86] have the potential to profit from these data. By design, *Open Humans* works similar to a PIMS, as it allows individuals to bundle and collect their personal data from external sources. Like other PIMS, *Open Humans* is likely to profit from any increase in data export functions that occur, e.g. due to the GDPR.

While the availability of data export functions is a necessary condition for making PIMS work, it alone is not sufficient. PIMS need to support the data import on their end, either by supporting the file types or by offering support for the application programming interfaces (APIs) of the external services. As file formats and APIs are not static, but can change over time, especially in case of popular services [87], a significant amount of effort is needed to keep data import functions into PIMS up to date. This cost keeps accumulating and increasing as the number of supported data imports keeps increasing. The modular, project-based nature of *Open Humans* allows the distribution of the workload of keeping integrations up to date, as data importers can be provided by any third party. Existing data imports on *Open Humans* already demonstrate this capability: Both the *Nightscout* as well as the *Apple HealthKit* data importer are examples of this. In case of *Nightscout*, members of the diabetes community themselves built and maintain the data import into *Open Humans* to power their own data commons that overlays the *Open Humans* data storage. The *HealthKit* import application was written by an individual *Open Humans* member who wanted to add support for adding their own data.

Enabling individual-centric research & citizen science

Open Humans provides several benefits for citizen science efforts and individual researchers who do not work in academia. The *OpenAPS* and *Nightscout Data Commons* highlighted in the results are prime examples of how Open Humans can enable such participant-lead research.

To enable research done by non-traditional researchers, the project creation workflow of Open Humans includes information for project leaders about informed consent and other key considerations. It encourages project administrators to be clear about both data management and security in a thorough community guide `https://www.openhumans.org/community-guidelines/#project`. This guide includes best practice guidelines for data security as well as details on how to communicate to participants which data access is being requested and why. It's emphasis on plain language and consideration of all of these elements, can result in an increased quality of the informed consent.

To further the community's ownership in the *Open Humans* platform, the community is involved in the governance of the ecosystem. On a high level the community gets to elect parts of the Board members of the foundation that is running Open Humans, enabling them to take direct influence on the larger direction of the platform. Furthermore, members of Open Humans are asked to vote on the approval of new projects that want to start on the platform, giving members the chance to review upcoming studies.

Summary

Here we present *Open Humans*, an active online platform for personal data aggregation and data sharing that enables citizen science and traditional academic science alike. By centering the data sharing decision on individual members it offers an ethical way of doing personal data-based research and furthermore enables individuals to better utilize their own data.

Methods

The primary Open Humans web application, as well as data source *Projects* maintained directly by *Open Humans*, are written in Python 3 using the Django web framework. API endpoints, JSON and HTML data serialization, and OAuth2 authorization are managed by the *Django REST Framework* and *Django OAuth Toolkit* libraries. Web apps are deployed on *Heroku* and use *Amazon S3* for file storage. The *Personal Data Notebooks* JupyterHub project is deployed via *Google Cloud Platform*.

Two Python packages have been developed and distributed in the *Python Package Index* to facilitate interactions with our API: (1) *open-humans-api* provides Python functions for API endpoints, as well as command line tools for performing many standard API operations, (2) *django-open-humans* provides a reusable Django module for using *Open Humans* OAuth2 and API features.

Availability of source code and requirements

- Project name: Open Humans
- Project home page: http://www.openhumans.org
- Operating system(s): Platform independent
- Programming language: Python3
- Other requirements: full list on GitHub https://github.com/openhumans/open-humans/
- License: MIT

- Project name: Open Humans API
- Project home page: https://open-humans-api.readthedocs.io/en/latest/
- Operating system(s): Platform independent
- Programming language: Python3
- Other requirements: full list on GitHub https://github.com/openhumans/open-humans-api
- License: MIT

- Project name: Django Open Humans
- Project home page: https://github.com/OpenHumans/django-open-humans
- Operating system(s): Platform independent
- Programming language: Python3
- Other requirements: full list on GitHub
- License: MIT

Declarations

List of abbreviations

CGM: Continuous Glucose Monitor DTC: Direct to Consumer GDPR General Data Protection Regulation IRB: Institutional Review Board PIMS: Personal information management systems QoL: Quality of Life

Ethical Approval

Not applicable

Consent for publication

Not applicable

Competing Interests

BGT is supported by a fellowship from *Open Humans Foundation*, which operates *Open Humans*. MPB is independently funded for full time work at *Open Humans Foundation* as Executive Director and President.

Funding

The development and operation of Open Humans has been supported through grants from the Robert Wood Johnson Foundation, John S. and James L. Knight Foundation, and Shuttleworth Foundation.

Author's Contributions

BGT: Conceptualization, Data curation, Investigation, Methodology, Project administration, Software, Supervision, Writing – original draft, Writing – review & editing TH: Methodology, Resources, Software DL: Data curation, Formal analysis, Validation, Writing – original draft, Writing – review & editing VE: Data curation, Formal analysis, Investigation, Validation, Visualization, Writing – original draft, Writing – review & editing KA: Data curation, Software, Validation, Writing – original draft, Writing – review & editing OS: Investigation, Validation, Writing – review & editing ON: Investigation, Validation BG: Data curation, Resources, Software, Validation MD: Software, Writing – review & editing JB: Conceptualization, Funding acquisition, Resources, Investigation, Project administration, Supervision MPB: Conceptualization, Data curation, Funding acquisition, Investigation, Methodology, Project administration, Resources, Software, Supervision, Writing – original draft, Writing – review & editing

Acknowledgements

The authors would like to thank all members of the Open Humans community for their diverse contributions to Open Humans: Developing the process as well as platforms that link to Open Humans, sharing their personal data, advancing public knowledge sources, being active community members.

In this spirit, this manuscript was written as a community project done by and with Open Humans members following an open call for contributions.

In particular, the authors would like to thank Rosy Gupta, Manaswini Das, Jasmine Tamak and Tarannum Khan. They made valuable contributions as summer interns with Open Hu-

mans through the Outreachy internship program. The authors are grateful to Mike Escalante, who contributed in software development as well as mentoring for Outreachy.

References

1. McCormick TH, Lee H, Cesare N, Shojaie A, Spiro ES. Using Twitter for Demographic and Social Science Research: Tools for Data Collection and Processing. Sociological Methods & Research 2015 oct;46(3):390–421. https://doi.org/10.1177/0049124115605339.
2. Özdemir V, Dove ES, Gürsoy UK, Şardaş S, Yıldırım A, Yılmaz ŞG, et al. Personalized medicine beyond genomics: alternative futures in big data—proteomics, environtome and the social proteome. Journal of Neural Transmission 2015 dec;124(1):25–32. https://doi.org/10.1007/s00702-015-1489-y.
3. Athey S. Beyond prediction: Using big data for policy problems. Science 2017 feb;355(6324):483–485. https://doi.org/10.1126/science.aal4321.
4. Cappella JN. Vectors into the Future of Mass and Interpersonal Communication Research: Big Data, Social Media, and Computational Social Science. Human Communication Research 2017 jun;43(4):545–558. https://doi.org/10.1111/hcre.12114.
5. Collins FS, Varmus H. A New Initiative on Precision Medicine. New England Journal of Medicine 2015 feb;372(9):793–795. https://doi.org/10.1056/nejmp1500523.
6. Chhibber A, Kroetz DL, Tantisira KG, McGeachie M, Cheng C, Plenge R, et al. Genomic architecture of pharmacological efficacy and adverse events. Pharmacogenomics 2014 dec;15(16):2025–2048. https://doi.org/10.2217/pgs.14.144.
7. Kummar S, Williams PM, Lih CJ, Polley EC, Chen AP, Rubinstein LV, et al. Application of Molecular Profiling in Clinical Trials for Advanced Metastatic Cancers. JNCI Journal of the National Cancer Institute 2015 feb;107(4):djv003–djv003. https://doi.org/10.1093/jnci/djv003.
8. Dilsizian SE, Siegel EL. Artificial Intelligence in Medicine and Cardiac Imaging: Harnessing Big Data and Advanced Computing to Provide Personalized Medical Diagnosis and Treatment. Current Cardiology Reports 2013 dec;16(1). https://doi.org/10.1007/s11886-013-0441-8.
9. Moon H, Ahn H, Kodell RL, Baek S, Lin CJ, Chen JJ. Ensemble methods for classification of patients for personalized medicine with high-dimensional data. Artificial Intelligence in Medicine 2007 nov;41(3):197–207. https://doi.org/10.1016/j.artmed.2007.07.003.
10. Kohane IS. Ten things we have to do to achieve precision medicine. Science 2015 jul;349(6243):37–38. https://doi.org/10.1126/science.aab1328.
11. Wetterstrand LA, DNA Sequencing Costs: Data; 2018. https://www.genome.gov/sequencingcostsdata/.
12. Swan M. Emerging Patient-Driven Health Care Models: An Examination of Health Social Networks, Consumer Personalized Medicine and Quantified Self-Tracking. International Journal of Environmental Research and Public Health 2009 feb;6(2):492–525. https://doi.org/10.3390/ijerph6020492.
13. Gay V, Leijdekkers P. Bringing Health and Fitness Data Together for Connected Health Care: Mobile Apps as Enablers of Interoperability. Journal of Medical Internet Research 2015 nov;17(11):e260. https://doi.org/10.2196/jmir.5094.
14. Corpas M, Valdivia-Granda W, Torres N, Greshake B, Coletta A, Knaus A, et al. Crowdsourced direct-to-consumer genomic analysis of a family quartet. BMC Genomics 2015 nov;16(1). https://doi.org/10.1186/s12864-015-1973-7.
15. Regalado A, 2017 was the year consumer DNA testing blew up; 2018. https://www.technologyreview.com/s/610233/2017-was-the-year-consumer-dna-testing-blew-up/.
16. Khan R, Mittelman D. Consumer genomics will change your life, whether you get tested or not. Genome Biology 2018 aug;19(1). https://doi.org/10.1186/s13059-018-1506-1.
17. EMC, The digital universe: Driving data growth in healthcare; 2014. https://web.archive.org/web/20180525094214/https://www.emc.com/analyst-report/digital-universe-healthcare-vertical-report-ar.pdf.
18. Rozenblum R, Bates DW. Patient-centred healthcare, social media and the internet: the perfect storm? BMJ Quality & Safety 2013 feb;22(3):183–186. https://doi.org/10.1136/bmjqs-2012-001744.
19. DeAngelis S, Patient Monitoring, Big Data, and the Future of Healthcare; 2014. https://www.wired.com/insights/2014/08/patient-monitoring-big-data-future-healthcare/.
20. Weber GM, Mandl KD, Kohane IS. Finding the Missing Link for Big Biomedical Data. JAMA 2014 may;https://doi.org/10.1001/jama.2014.4228.
21. Carbon S, Champieux R, McMurry J, Winfree L, Wyatt LR, Haendel M. A Measure of Open Data: A Metric and Analysis of Reusable Data Practices in Biomedical Data Resources 2018 mar;https://doi.org/10.1101/282830.
22. Blasimme A, Fadda M, Schneider M, Vayena E. Data Sharing For Precision Medicine: Policy Lessons And Future Directions. Health Affairs 2018 may;37(5):702–709. https://doi.org/10.1377/hlthaff.2017.1558.
23. Kossmann D, Brand A, Hafen E. Health Data Cooperatives – Citizen Empowerment. Methods of Information in Medicine 2014;53(02):82–86. https://doi.org/10.3414/me13-02-0051.
24. Tenopir C, Allard S, Douglass K, Aydinoglu AU, Wu L, Read E, et al. Data Sharing by Scientists: Practices and Perceptions. PLoS ONE 2011 jun;6(6):e21101. https://doi.org/10.1371/journal.pone.0021101.
25. Lye CT, Forman HP, Gao R, Daniel JG, Hsiao AL, Mann MK, et al. Assessment of US Hospital Compliance With Regulations for Patients' Requests for Medical Records. JAMA Network Open 2018 oct;1(6):e183014. https://doi.org/10.1001/jamanetworkopen.2018.3014.
26. Blumenthal D, Tavenner M. The "Meaningful Use" Regulation for Electronic Health Records. New England Journal of Medicine 2010 aug;363(6):501–504. https://doi.org/10.1056/nejmp1006114.
27. Hert PD, Papakonstantinou V, Malgieri G, Beslay L, Sanchez I. The right to data portability in the GDPR: Towards user-centric interoperability of digital services. Computer Law & Security Review 2018 apr;34(2):193–203. https://doi.org/10.1016/j.clsr.2017.10.003.
28. Recommendation on Return of Individual Research Results; 2016. https://www.hhs.gov/ohrp/sachrp-committee/recommendations/attachment-b-return-individual-research-results/index.html.
29. Wong CA, Hernandez AF, Califf RM. Return of Research Results to Study Participants. JAMA 2018 aug;320(5):435. https://doi.org/10.1001/jama.2018.7898.
30. Mason PH. The Ethics of Biomedical Big Data. Journal of Bioethical Inquiry 2017 oct;14(4):571–574. https://doi.org/10.1007/s11673-017-9812-y.
31. Ross MW, Iguchi MY, Panicker S. Ethical aspects of data sharing and research participant protections. American Psychologist 2018 feb;73(2):138–145. https://doi.org/10.1037/amp0000240.

32. Haeusermann T, Greshake B, Blasimme A, Irdam D, Richards M, Vayena E. Open sharing of genomic data: Who does it and why? PLOS ONE 2017 may;12(5):e0177158. https://doi.org/10.1371/journal.pone.0177158.
33. Wang S, Jiang X, Singh S, Marmor R, Bonomi L, Fox D, et al. Genome privacy: challenges, technical approaches to mitigate risk, and ethical considerations in the United States. Annals of the New York Academy of Sciences 2016 sep;1387(1):73–83. https://doi.org/10.1111/nyas.13259.
34. Evans BJ. Power to the People: Data Citizens in the Age of Precision Medicine. Vanderbilt J Entertain Technol Law 2017;19(2):243–265.
35. Samerski S. Individuals on alert: digital epidemiology and the individualization of surveillance. Life Sciences, Society and Policy 2018 jun;14(1). https://doi.org/10.1186/s40504-018-0076-z.
36. Cox J, 70,000 OkCupid Users Just Had Their Data Published; 2016. http://motherboard.vice.com/read/70000-okcupid-users-just-had-their-data-published.
37. Zimmer M. "But the data is already public": on the ethics of research in Facebook. Ethics and Information Technology 2010 jun;12(4):313–325. https://doi.org/10.1007/s10676-010-9227-5.
38. Zook M, Barocas S, danah boyd, Crawford K, Keller E, Gangadharan SP, et al. Ten simple rules for responsible big data research. PLOS Computational Biology 2017 mar;13(3):e1005399. https://doi.org/10.1371/journal.pcbi.1005399.
39. Jouhki J, Lauk E, Penttinen M, Sormanen N, Uskali T. Facebook's Emotional Contagion Experiment as a Challenge to Research Ethics. Media and Communication 2016 oct;4(4):75. https://doi.org/10.17645/mac.v4i4.579.
40. Hunter D, Evans N. Facebook emotional contagion experiment controversy. Research Ethics 2016 jan;12(1):2–3. https://doi.org/10.1177/1747016115626341.
41. Flick C. Informed consent and the Facebook emotional manipulation study. Research Ethics 2015 aug;12(1):14–28. https://doi.org/10.1177/1747016115599568.
42. Bruns A, Facebook Shuts the Gate after the Horse Has Bolted, and Hurts Real Research in the Process; 2018. https://medium.com/@Snurb/facebook-research-data-18662cf2cacb.
43. Golder S, Ahmed S, Norman G, Booth A. Attitudes Toward the Ethics of Research Using Social Media: A Systematic Review. Journal of Medical Internet Research 2017 jun;19(6):e195. https://doi.org/10.2196/jmir.7082.
44. Pocock MJO, Tweddle JC, Savage J, Robinson LD, Roy HE. The diversity and evolution of ecological and environmental citizen science. PLOS ONE 2017 apr;12(4):e0172579. https://doi.org/10.1371/journal.pone.0172579.
45. Vayena E, Tasioulas J. "We the Scientists": a Human Right to Citizen Science. Philosophy & Technology 2015 jun;28(3):479–485. https://doi.org/10.1007/s13347-015-0204-0.
46. McKinley DC, Miller-Rushing AJ, Ballard HL, Bonney R, Brown H, Cook-Patton SC, et al. Citizen science can improve conservation science, natural resource management, and environmental protection. Biological Conservation 2017 apr;208:15–28. https://doi.org/10.1016/j.biocon.2016.05.015.
47. Conrad CC, Hilchey KG. A review of citizen science and community-based environmental monitoring: issues and opportunities. Environmental Monitoring and Assessment 2010 jul;176(1-4):273–291. https://doi.org/10.1007/s10661-010-1582-5.
48. Zevin M, Coughlin S, Bahaadini S, Besler E, Rohani N, Allen S, et al. Gravity Spy: integrating advanced LIGO detector characterization, machine learning, and citizen science. Classical and Quantum Gravity 2017 feb;34(6):064003. https://doi.org/10.1088/1361-6382/aa5cea.
49. Haklay M. Citizen Science and Volunteered Geographic Information: Overview and Typology of Participation. In: Crowdsourcing Geographic Knowledge Springer Netherlands; 2012.p. 105–122. https://doi.org/10.1007/978-94-007-4587-2_7.
50. Dickinson H, Fortson L, Lintott C, Scarlata C, Willett K, Bamford S, et al. Galaxy Zoo: Morphological Classification of Galaxy Images from the Illustris Simulation. The Astrophysical Journal 2018 feb;853(2):194. https://doi.org/10.3847/1538-4357/aaa250.
51. Khatib F, Cooper S, Tyka MD, Xu K, Makedon I, Popovic Z, et al. Algorithm discovery by protein folding game players. Proceedings of the National Academy of Sciences 2011 nov;108(47):18949–18953. https://doi.org/10.1073/pnas.1115898108.
52. Ranard BL, Ha YP, Meisel ZF, Asch DA, Hill SS, Becker LB, et al. Crowdsourcing—Harnessing the Masses to Advance Health and Medicine, a Systematic Review. Journal of General Internal Medicine 2013 jul;29(1):187–203. https://doi.org/10.1007/s11606-013-2536-8.
53. Rowbotham S, McKinnon M, Leach J, Lamberts R, Hawe P. Does citizen science have the capacity to transform population health science? Critical Public Health 2017 nov;p. 1–11. https://doi.org/10.1080/09581596.2017.1395393.
54. Mader LB, Harris T, Kläger S, Wilkinson IB, Hiemstra TF. Inverting the patient involvement paradigm: defining patient led research. Research Involvement and Engagement 2018 jul;4(1). https://doi.org/10.1186/s40900-018-0104-4.
55. Vayena E, Brownsword R, Edwards SJ, Greshake B, Kahn JP, Ladher N, et al. Research led by participants: a new social contract for a new kind of research. Journal of Medical Ethics 2015 mar;42(4):216–219. https://doi.org/10.1136/medethics-2015-102663.
56. Katapally TR, Bhawra J, Leatherdale ST, Ferguson L, Longo J, Rainham D, et al. The SMART Study, a Mobile Health and Citizen Science Methodological Platform for Active Living Surveillance, Integrated Knowledge Translation, and Policy Interventions: Longitudinal Study. JMIR Public Health and Surveillance 2018 mar;4(1):e31. https://doi.org/10.2196/publichealth.8953.
57. Wicks P, Vaughan TE, Massagli MP, Heywood J. Accelerated clinical discovery using self-reported patient data collected online and a patient-matching algorithm. Nature Biotechnology 2011 apr;29(5):411–414. https://doi.org/10.1038/nbt.1837.
58. McDonald D, Hyde E, Debelius JW, Morton JT, Gonzalez A, Ackermann G, et al. American Gut: an Open Platform for Citizen Science Microbiome Research. mSystems 2018;3(3). https://msystems.asm.org/content/3/3/e00031-18.
59. McGowan ML, Choudhury S, Juengst ET, Lambrix M, Settersten RA, Fishman JR. "Let's pull these technologies out of the ivory tower": The politics, ethos, and ironies of participant-driven genomic research. BioSocieties 2017 mar;12(4):494–519. https://doi.org/10.1057/s41292-017-0043-6.
60. Levitan B, Getz K, Eisenstein EL, Goldberg M, Harker M, Hesterlee S, et al. Assessing the Financial Value of Patient Engagement. Therapeutic Innovation & Regulatory Science 2017 jul;52(2):220–229. https://doi.org/10.1177/2168479017716715.
61. Swan M. The Quantified Self: Fundamental Disruption in Big Data Science and Biological Discovery. Big Data 2013 jun;1(2):85–99. https://doi.org/10.1089/big.2012.0002.
62. Swan M. Health 2050: The Realization of Personalized Medicine through Crowdsourcing, the Quantified Self, and the Participatory Biocitizen. Journal of Personalized

Medicine 2012 sep;2(3):93–118. https://doi.org/10.3390/jpm2030093.
63. Yuan J, Gordon A, Speyer D, Aufrichtig R, Zielinski D, Pickrell J, et al. DNA.Land is a framework to collect genomes and phenomes in the era of abundant genetic information. Nature Genetics 2018 jan;50(2):160–165. https://doi.org/10.1038/s41588-017-0021-8.
64. Greshake B, Bayer PE, Rausch H, Reda J. openSNP–A Crowdsourced Web Resource for Personal Genomics. PLoS ONE 2014 mar;9(3):e89204. https://doi.org/10.1371/journal.pone.0089204.
65. Ball MP, Thakuria JV, Zaranek AW, Clegg T, Rosenbaum AM, Wu X, et al. A public resource facilitating clinical use of genomes. Proceedings of the National Academy of Sciences 2012 jul;109(30):11920–11927. https://doi.org/10.1073/pnas.1201904109.
66. Lewis D, and SL. Real-World Use of Open Source Artificial Pancreas Systems. Journal of Diabetes Science and Technology 2016 aug;10(6):1411–1411. https://doi.org/10.1177/1932296816665635.
67. Lewis DM, Ball MP. OpenAPS Data Commons on Open Humans 2017 9;https://figshare.com/articles/OpenAPS_Data_Commons_on_Open_Humans/5428498.
68. Lewis DM, Leibrand S, Street TJ, Phatak SS. Detecting Insulin Sensitivity Changes for Individuals with Type 1 Diabetes. Diabetes 2018 may;67(Supplement 1):79–LB. https://doi.org/10.2337/db18-79-lb.
69. Das S, Forer L, Schönherr S, Sidore C, Locke AE, Kwong A, et al. Next-generation genotype imputation service and methods. Nature Genetics 2016 Aug;48:1284 EP –. http://dx.doi.org/10.1038/ng.3656.
70. Lemieux Perreault LP, Legault MA, Asselin G, Dubé MP. genipe: an automated genome-wide imputation pipeline with automatic reporting and statistical tools. Bioinformatics 2016;32(23):3661–3663. http://dx.doi.org/10.1093/bioinformatics/btw487.
71. Consortium TGP, Auton A, Abecasis GR, Altshuler (Co-Chair) DM, Durbin (Co-Chair) RM, Bentley DR, et al. A global reference for human genetic variation. Nature 2015 Sep;526:68 EP –. http://dx.doi.org/10.1038/nature15393, article.
72. WH D, CE D, RC B. Chronic disease prevention: Tobacco avoidance, physical activity, and nutrition for a healthy start. JAMA 2016;316(16):1645–1646. +http://dx.doi.org/10.1001/jama.2016.14370.
73. Westendorf L, Shaer O, Pollalis C, Verish C, Nov O, Ball MP. Exploring Genetic Data Across Individuals: Design and Evaluation of a Novel Comparative Report Tool. Journal of Medical Internet Research 2018 sep;20(9):e10297. https://doi.org/10.2196/10297.
74. Kluyver T, Ragan-Kelley B, Pérez F, Granger B, Bussonnier M, Frederic J, et al. Jupyter Notebooks – a publishing format for reproducible computational workflows. In: Loizides F, Schmidt B, editors. Positioning and Power in Academic Publishing: Players, Agents and Agendas IOS Press; 2016. p. 87 – 90.
75. Wilkinson MD, Dumontier M, Aalbersberg IJ, Appleton G, Axton M, Baak A, et al. The FAIR Guiding Principles for scientific data management and stewardship. Scientific Data 2016 mar;3:160018. https://doi.org/10.1038/sdata.2016.18.
76. Kullenberg C, Kasperowski D. What Is Citizen Science? – A Scientometric Meta-Analysis. PLOS ONE 2016 jan;11(1):e0147152. https://doi.org/10.1371/journal.pone.0147152.
77. Power to the Patients: Co-design of Community-based Research; 2018. http://blogs.plos.org/blog/2018/08/09/power-to-the-patients-co-design-of-community-based-research/.
78. Schwartz PH, Caine K, Alpert SA, Meslin EM, Carroll AE, Tierney WM. Patient Preferences in Controlling Access to Their Electronic Health Records: a Prospective Cohort Study in Primary Care. Journal of General Internal Medicine 2014 dec;30(S1):25–30. https://doi.org/10.1007/s11606-014-3054-z.
79. Grando MA, Murcko A, Mahankali S, Saks M, Zent M, Chern D, et al. A Study to Elicit Behavioral Health Patients' and Providers' Opinions on Health Records Consent. The Journal of Law, Medicine & Ethics 2017 jun;45(2):238–259. https://doi.org/10.1177/1073110517720653.
80. Caine K, Hanania R. Patients want granular privacy control over health information in electronic medical records. Journal of the American Medical Informatics Association 2013 jan;20(1):7–15. https://doi.org/10.1136/amiajnl-2012-001023.
81. Nati M, Mayer S, Capossele A, Missier P. Toward Trusted Open Data and Services. Internet Technology Letters 2018 jul;p. e69. https://doi.org/10.1002/itl2.69.
82. The Global Alliance for Genomics and Health. A federated ecosystem for sharing genomic, clinical data. Science 2016 jun;352(6291):1278–1280. https://doi.org/10.1126/science.aaf6162.
83. Althoff T. Population-Scale Pervasive Health. IEEE Pervasive Computing 2017 oct;16(4):75–79. https://doi.org/10.1109/mprv.2017.3971134.
84. Allard T, Bouadi T, Duguépéroux J, Sans V. From Self-data to Self-preferences: Towards Preference Elicitation in Personal Information Management Systems. In: Personal Analytics and Privacy. An Individual and Collective Perspective Springer International Publishing; 2017.p. 10–16. https://doi.org/10.1007/978-3-319-71970-2_2.
85. Rumbold JMM, Pierscionek B. The Effect of the General Data Protection Regulation on Medical Research. Journal of Medical Internet Research 2017 feb;19(2):e47. https://doi.org/10.2196/jmir.7108.
86. Quinn P. Is the GDPR and Its Right to Data Portability a Major Enabler of Citizen Science? Global Jurist 2018 jun;18(2). https://doi.org/10.1515/gj-2018-0021.
87. Xavier L, Brito A, Hora A, Valente MT. Historical and impact analysis of API breaking changes: A large-scale study. In: 2017 IEEE 24th International Conference on Software Analysis, Evolution and Reengineering (SANER) IEEE; 2017. https://doi.org/10.1109/saner.2017.7884616.
