# Supplementary material for: Open Humans: A platform for participant-centered research and personal data exploration
Source: Gigascience. 2019 Jun 26;8(6):giz076. doi: 10.1093/gigascience/giz076 (PMC6593360; doi:10.1093/gigascience/giz076)
Supplement: giz076_GIGA-D-18-00451_Revision_2 [file giz076_giga-d-18-00451_revision_2.pdf]

GigaScience

Open Humans: A platform for participant-centered research and personal data exploration

--Manuscript Draft--

**Manuscript Number:**	GIGA-D-18-00451R2
**Full Title:**	Open Humans: A platform for participant-centered research and personal data exploration
**Article Type:**	Review
**Funding Information:**	Robert Wood Johnson Foundation (NA) — Not applicable<br>John S. and James L. Knight Foundation (NA) — Not applicable
**Abstract:**	Background: Many aspects of our lives are now digitized and connected to the internet. As a result, individuals are now creating and collecting more personal data than ever before. This offers an unprecedented chance for human-participant research ranging from the social sciences to precision medicine. With this potential wealth of data come practical problems (such as how to merge data streams from various sources),as well as ethical problems (such as how to best balance risks and benefits when enabling personal data sharing by individuals).<br>Results: To begin to address these problems in real time, we present Open Humans, a community-based platform that enables personal data collections across data streams, giving individuals more personal data access and control of sharing authorizations, and enabling academic research as well as patient-led projects. We showcase data streams that Open Humans combines (e.g. personal genetic data, wearable activity monitors, GPS location records and continuous glucose monitor data), along with use cases of how the data facilitates various projects.<br>Conclusions: Open Humans highlights how a community-centric ecosystem can be used to aggregate personal data from various sources as well as how these data can be used by academic and citizen scientists through practical, iterative approaches to sharing that strive to balance considerations with participant autonomy, inclusion, and privacy.
**Corresponding Author:**	Bastian Greshake Tzovaras, Ph.D<br>E O Lawrence Berkeley National Laboratory<br>UNITED STATES
**Corresponding Author Secondary Information:**	
**Corresponding Author's Institution:**	E O Lawrence Berkeley National Laboratory
**Corresponding Author's Secondary Institution:**	
**First Author:**	Bastian Greshake Tzovaras, Ph.D
**First Author Secondary Information:**	
**Order of Authors:**	Bastian Greshake Tzovaras, Ph.D
	Misha Angrist
	Kevin Arvai
	Mairi Dulaney
	Vero Estrada-Galinanes
	Beau Gunderson
	Tim Head
	Dana Lewis
	Oded Nov

	Orit Shaer
	Athina Tzovara
	Jason Bobe
	Mad Price Ball
**Order of Authors Secondary Information:**	
**Response to Reviewers:**	We have addressed the additional comments of the reviewers.<br><br>The LaTeX source with tracked changes for the manuscript can be viewed at https://www.overleaf.com/read/kybdrqszgpzs<br><br>For the individual comments:<br><br>1) P3C2P2. Under Results...Second sentence could be improved. Try this "Platform members import data about themselves from various sources into their Open Humans account. They can then explore their aggregated data and share it with citizen scientists and academic researchers."<br>This is a great idea, we have incorporated this change to improve the sentence.<br><br>2) P4C2P2. Is that on a project by project basis or global across all projects the member has joined?<br>This is on a project-by-project basis and we have rephrased this to make it clearer.<br><br>3) P9C1P2. The sentence "While Article 20 does not..." needs to be cleaned up.<br>Very good point, we have cleaned it up.<br><br>4) P9C1P4. participant-led or -lead?<br>This should have been participant-led and we have fixed it.<br><br>We also want to thank the reviewers for all the time they have put into reviewing our manuscript and the extremely useful feedback they have given. We feel that the manuscript has improved dramatically thanks to their input. We would like to ask the reviewers whether they would like to be listed with their names in the acknowledgement? Given all their work, we would like to fully credit them for their work if they agree.
**Additional Information:**	
**Question**	**Response**
Are you submitting this manuscript to a special series or article collection?	No
**Experimental design and statistics**<br><br>Full details of the experimental design and statistical methods used should be given in the Methods section, as detailed in our Minimum Standards Reporting Checklist. Information essential to interpreting the data presented should be made available in the figure legends.<br><br>Have you included all the information requested in your manuscript?	Yes
**Resources**	Yes

A description of all resources used, including antibodies, cell lines, animals and software tools, with enough information to allow them to be uniquely identified, should be included in the Methods section. Authors are strongly encouraged to cite Research Resource Identifiers (RRIDs) for antibodies, model organisms and tools, where possible.<br><br>Have you included the information requested as detailed in our Minimum Standards Reporting Checklist?	
**Availability of data and materials**<br><br>All datasets and code on which the conclusions of the paper rely must be either included in your submission or deposited in publicly available repositories (where available and ethically appropriate), referencing such data using a unique identifier in the references and in the “Availability of Data and Materials” section of your manuscript.<br><br>Have you have met the above requirement as detailed in our Minimum Standards Reporting Checklist?	Yes

Powered by Editorial Manager® and ProduXion Manager® from Aries Systems Corporation

Manuscript

Click here to view linked References

Click here to access/download;Manuscript;open_humans_manuscript.pdf

GigaScience, 2017, 1–13

doi: xx.xxxx/xxxx
Manuscript in Preparation
Paper

PAPER

Open Humans: A platform for participant-centered research and personal data exploration

Bastian Greshake Tzovaras[1,2,*], Misha Angrist[3,†], Kevin Arvai[,†], Mairi Dulaney[1,†], Vero Estrada-Galiñanes[4,5,†], Beau Gunderson[,†], Tim Head[6,†], Dana Lewis[7,†], Oded Nov[8,†], Orit Shaer[9,†], Athina Tzovara[10,11,†], Jason Bobe[12] and Mad Price Ball[1,*]

[1]Open Humans Foundation, USA and [2]Lawrence Berkeley National Laboratory, Berkeley, CA, USA and [3]Social Science Research Institute, Duke University, Durham, NC, USA and [4]QoL Lab, Department of Computer Science, University of Copenhagen, Denmark and [5]IDE, University of Stavanger, Norway and [6]Wild Tree Tech, Switzerland and [7]OpenAPS, Seattle, WA, USA and [8]Tandon School of Engineering, New York University, New York, USA and [9]Wellesley College, Wellesley, MA, USA and [10]Helen Wills Neuroscience Institute, University of California, Berkeley, CA, USA and [11]Institute of Computer Science, University of Bern, Switzerland and [12]Institute for Next Generation Healthcare, Icahn School of Medicine at Mount Sinai, NY, USA

*bgreshake@gmail.com; mpball@gmail.com
†Authors contributed equally

Abstract

Background: Many aspects of our lives are now digitized and connected to the internet. As a result, individuals are now creating and collecting more personal data than ever before. This offers an unprecedented chance for human-participant research ranging from the social sciences to precision medicine. With this potential wealth of data come practical problems (such as how to merge data streams from various sources), as well as ethical problems (such as how to best balance risks and benefits when enabling personal data sharing by individuals). **Results:** To begin to address these problems in real time, we present Open Humans, a community-based platform that enables personal data collections across data streams, giving individuals more personal data access and control of sharing authorizations, and enabling academic research as well as patient-led projects. We showcase data streams that Open Humans combines (e.g. personal genetic data, wearable activity monitors, GPS location records and continuous glucose monitor data), along with use cases of how the data facilitates various projects. **Conclusions:** Open Humans highlights how a community-centric ecosystem can be used to aggregate personal data from various sources as well as how these data can be used by academic and citizen scientists through practical, iterative approaches to sharing that strive to balance considerations with participant autonomy, inclusion, and privacy.

Key words: Personal Data; Crowdsourcing; Citizen Science; Database; Open Data ; Participatory Science; Peer Production

Background

Research involving human participants, from biomedical and health research to social sciences studies, is experiencing rapid changes. The rise of electronic records, online platforms, and data from devices contribute to a sense that these collected data can change how research in these fields is performed [1, 2, 3, 4]

Among the impacted disciplines is precision medicine –

Compiled on: June 1, 2019.
Draft manuscript prepared by the author.

which takes behavioral, environmental, and genetic factors into account and has become a vision for health-care in the United States [5]. By taking individual parameters into account, precision medicine aims to improve health outcomes, for example by optimizing drugs based on a patient's genetic makeup [6, 7].

Access to large-scale data sets, along with availability of appropriate methods to analyze these data [8, 9], is often described as a major prerequisite for the success of precision medicine [10]. Falling costs for large-scale, individualized analyses such as whole-genome sequencing [11] have already helped facilitate both research in precision medicine and its adoption. In addition, an increasing number of patients and healthy individuals are collecting health-related data outside traditional healthcare, for example through smartphones and wearable devices [12, 13] or through direct-to-consumer (DTC) genetic testing [14].

Indeed, at least 12-17 million individuals have taken a DTC genetic test [15, 16], while more than 25 million such tests have been purchased [17]. Meanwhile, it is estimated that by 2020 over two exabytes of storage will be needed for health care data [18] alone. Furthermore, data from social network sites like Facebook and Twitter are increasingly likely targets for medical data mining [19]. Additionally, more data is becoming available from personal medical devices, both in real-time and for retrospective analyses [20].

These changes to research and medical practice bring with them a number of challenges, including the problems of data silos, ethical data sharing, and participant involvement. A participant-centered approach to personal data aggregation, sharing, and research has the potential to address these issues. To achieve this, we created *Open Humans* as a digital ecosystem designed to facilitate individual data aggregation across data sources, granular management of data sharing, and co-created research.

Data Silos

To fully realize the promises of these large personal data collections, not only in precision medicine but all fields of research, access to both big data and smaller data sources is needed, as is the ability to tap into a variety of data streams and link these data [21, 10]. Data silos can hinder the merging and re-use of data by third parties for a number of reasons: they can be incompatible due to different data licenses [22] or inaccessible due to privacy, ethical, and regulatory concerns [23, 24, 25]. For example, the US National Human Genome Research Institute's Database of Genotypes and Phenotypes remains an underused resource because of logistical and regulatory/ethical oversight challenges for would-be users [26]. In addition to legal barriers, there are typically technical challenges in rendering data accessible, usable, and/or anonymized, and a data controller typically has incentives to seek compensation in return for these activities.

Beyond biomedical datasets, there are data from wearable devices, social media, and other data held by private companies, from which data exports are often not available. In other cases data access might be legally mandated, but the practical outcomes are mixed or in progress [27, 28], *e.g.*, for clinical health data in the United States as mandated by the 1996 *Health Insurance Portability and Accountability Act* and 2009 *Health Information Technology for Economic and Clinical Health Act* (HIPAA and HITECH Act), and for personal data in the European Union as mandated by rights to data access and data portability in the 2016 *General Data Protection Regulation* (GDPR) [29, 30]. In addition, within the context of research involving human participants, data access may be recommended [31] but not legally required, and as a result is not typically provided [32]. Data portability and easy access to research data by participating individuals could empower them to steer research in directions that affect their lives and health outcomes.

Ethical Data Re-Use

While the sharing and re-using of biomedical data can potentially transform medical care and medical research, it brings along a number of ethical considerations [33, 34]. In the field of human genetics, the ethics of sharing data has been extensively considered with respect to how research participants and patients can give informed consent for studies that carry risks of genetic discrimination, loss of privacy, and re-identification in publicly shared data [35, 36]. Due to access and portability issues, however, research with biomedical data is rarely driven by the individuals from whom the data came – and as a result, such research fails to give patients much power over how their data can be used [37]. For example, it is now abundantly clear that direct-to-consumer genetic testing customers routinely have their de-identified (but re-identifiable) data shared with third parties [38]. Open Humans seeks to be among the agents for change in this regard. Bottom-up research initiatives have included disease- and/or mutation-specific efforts [39, 40] and the development of platforms meant to allow participants to control data-sharing at a granular level [41]. Open Humans is meant to complement such initiatives and enable the creation of multiple "sandboxes" where both personal and biomedical data can be leveraged to help grow empirical knowledge and further downstream development of diagnostics and therapies.

Elsewhere, social media is also gaining importance in research as well as public health [42]. Differing perceptions on the sensitivity of social media data can lead to privacy concerns. For example, an analysis performed on 70,000 users of an online dating website, where private personal data was scraped by researchers and then publicly shared, caused a public outcry [43]. Such cases have sparked calls for caution in performing "big data" research with these new forms of personal data [44, 45].

Research that interacts with social media users raises additional concerns. For example, Facebook was widely criticized for an experiment to study emotional contagion among 700,000 of its users without their consent or debriefing, prompting discussion of the ethics of unregulated human subjects research and "A/B testing" by private entities [46, 47, 48]. And the 2018 disclosure of the Cambridge Analytica controversy, in which a private firm harvested information from 50 million Facebook users without their permission, led Facebook to tighten control over its application programming interfaces (APIs), turning it even more of a silo that does not allow for research to be done by outside researchers [49].

For the foreseeable future, researchers that re-use data from commercial sources will have to decide how to balance the interests of commercial data controllers, participants, and society. While there is no consensus on how research consent for existing personal data should be performed, we know that participants desire more granular abilities to manage data sharing: to decide who can and cannot see it, under what circumstances, and what can and cannot be done with it [50]. Such individual control will be especially critical in the sensitive context of precision medicine [24].

Participant Involvement

Citizen science mostly describes the involvement of volunteers in the data collection, analysis, and interpretation phases of

research projects [51], thus both supporting the research process itself and helping with public engagement. Furthermore, the Universal Declaration of Human Rights describes a broad human right to access science as a whole, implying a right to participate in all aspects of the scientific enterprise [52].

Traditionally, many participatory science projects have focused on the natural sciences, like natural resource management, environmental monitoring/protection, and astrophysics [53, 54, 55]. In many of these examples volunteers are asked to crowd-source and support scientists in the collection of data – e.g. by field observations or through sensors [56] or by performing human computation tasks such as classifying images [57] or generating folded protein-structures [58].

Analogous to the movement in other realms of citizen science, there is a growing movement toward more participant/patient involvement in research on humans, including in fields such as radiology, public health, psychology, and epidemiology [59, 60]. Patients often have a better understanding of their disease and needs than medical/research professionals [61, 62] and that patient involvement can help catalyze policy interventions [63]. Examples include the studies on amyotrophic lateral sclerosis initiated by *PatientsLikeMe* users [64], crowd-sourcing efforts like *American Gut* [65], and a variety of other *citizen genomics* efforts [66]. It is likely that involving patients in clinical research can not only help minimize cost but can lead to drugs being brought to market sooner [67].

Elsewhere, the *Quantified Self* movement, in which individuals perform self-tracking of biological, behavioral, or environmental information and design experiments with an *n=1* to learn about themselves [68], can be seen on this continuum of participant-led research [69]. By performing self-experiments and recording their own data, individuals can gain critical knowledge about themselves and the process of performing research. Analogous to the benefits of patient insights in clinical research, individuals engaged in self-tracking and personal data analysis have the potential to contribute their insights to a variety of other research areas.

A participant-centered approach to research

As shown above, substantially involving patients and participants in the research process has multiple benefits. Participants as primary data holders can help in breaking down walls among data silos and in aggregating and sharing personal data streams. Furthermore, by being involved in the research process and actively providing data, they can gain autonomy and can actively consent to their data being used, thus mitigating (but not eliminating) the likelihood of subsequent ethical concerns. Last but not least, enabling individuals to analyze and explore their own data, individually and collectively, can result in valuable feedback that helps researchers incorporate the needs, desires, and insights of participants.

In recent years a number of projects have started to explore both data donations and crowd-sourcing research with an extended involvement of participants. In genomics, both academic projects like *DNA.Land* [70] and community-driven projects like *openSNP* [71] are enabling crowdsourcing via personal genetic data set donations. Furthermore, the idea of *Health Data Cooperatives* that are communally run to manage access to health data has emerged [24].

However, most of these projects limit participants' involvement in the research process: a participant is limited, for example, to providing specific types of data for a specific data repository. Additionally, participants are rarely given an easy way to help in designing a study, let alone running their own.

To close these gaps we developed *Open Humans*, a community-based platform that enables its members to share a growing number of personal data types; participate in research projects and create their own; and facilitate the exploration of personal data by and for the individual member. *Open Humans* was initially conceived as an iteration of work with the Harvard Personal Genome Project [72]. Along with a description of the platform itself and its power and limitations, we present a set of examples on how the platform is already being used for academic and participant-led research projects.

Results

We designed *Open Humans* as a web platform with the goal of easily enabling connections to existing and newly created data sources and data (re-)using applications. Platform members import data about themselves from various sources into their Open Humans account. They can then explore their aggregated data and share it with projects from citizen scientists and academic researchers.

Design

In the center of the design are three main components: *Members*, *Projects* and *Data* objects. *Members* can join various *Projects* and authorize them to read *Data* that's stored in their account as well as write new *Data* for this *Member* (see Figure 1 for a dataflow diagram).

Projects

Projects are the primary way for *Members* to interact with *Open Humans*. *Projects* can be created by any *Member*. During project creation a prospective project lead must provide a description of their project and specify the access permissions they request from *Members* who decide to join. These may include:

Username By default projects do not get access to a *Member*'s username; each *Member* is identified with a random, unique identifier specific to that project. This way *Members* can join a project while being pseudonymous.

Data Access A *Project* may ask permission to read *Data* that have been deposited into a *Member*'s account by other projects. A project lead needs to specify to which existing projects' data they want to have access to and only this data will be shared with the new project.

Through the permission system, *Members* get a clear idea of the amount of *Data* they are sharing by joining a given *Project* and whether their username will be shared and with whom.

Furthermore, all *Projects* have the following permissions for any *Members* who have joined them: (1) they can send messages to *Members*, which are received as emails; and (2) they can upload new *Data* into the *Member* account. Thus, in addition to acting as potential data recipients, *Projects* are also the avenue by which *Data* is added to *Member* accounts.

Projects can be set up in two different ways: As an *On-site Project* or as an *OAuth2 Project*. The *OAuth2 Project* format offers a standard OAuth2 user authorization process commonly used to connect across web services. Projects that implement this can connect an Open Humans user to a separate mobile or web application, and can be fully automated. For projects that do not have separate applications, the *On-site Project* format allows the project to present "consent" or "terms of use" information within Open Humans, thereby minimizing the need for technical work on the part of a project. Both formats have access to APIs for performing data uploads, data access, and member messaging.

Projects also need to clearly signal whether they are a research study that is subject to ethical oversight by an Institutional Review Board (IRB) or equivalent, or whether they are

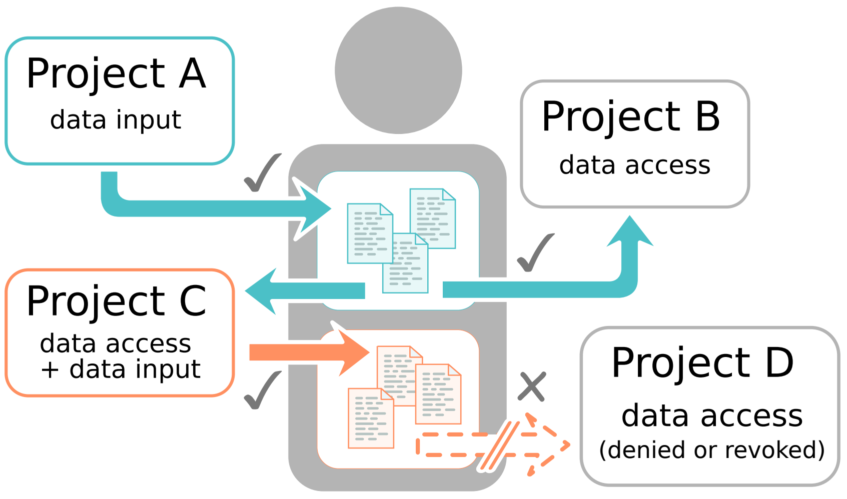

Figure 1. The Open Humans authorization flow. A *Member* (center) can join *Projects* and approve them to read or write *Data*. The *Member* approves *Project A* to deposit files (blue) into their account. They also approve *Project B* to read the files that *Project A* has deposited. Additionally, the *Member* approves *Project C* to both read the files of *Project A* and write new files. The *Member* declines to give access to their personal data to *Project D*.

not performing such research (i.e. not subject to this oversight). This allows for participant-led projects outside an academic research setting, provided *Members* see a notification alerting them to the absence of IRB oversight.

Thus, any Member can create a project in the site, at any time, and all APIs work immediately. However a *Project* will not be publicly listed for *Members* to see, and has a cap limiting the number of *Members* that may join. Public listing and unlimited usage is granted when a project is marked as "approved" following a community review process. *Projects* that have IRB oversight are required to provide documentation of IRB approval as part of this review process.

In summary, given the broad potential features available, a *Project* can cover anything from data import tools, to data processing tools, to research projects, to self-quantification projects that visualize and analyze a *Member*'s data.

Members

Members interact with *Projects* that are run on *Open Humans*. By joining projects that act as data uploaders, they can add specific *Data* into their Open Humans accounts. This is a way to connect external services: e.g. put their genetic data or activity tracking data into their *Open Humans* account. Once they have connected to relevant *Projects* that import their own data, *Members* can opt-in to joining additional *Projects* that they wish to grant access to their account's data.

As *Members* are able to selectively join *Projects*, they can elect which projects their *Data* should be shared with. Members may withdraw from a *Project* at any time. This results in immediate revocation of *Data*-sharing authorization for that *Project*, as well as a removal of *Data* upload and message permissions. *Projects* may also support data erasure requests upon withdrawal, and any remaining *Data* uploaded by a project may be retained or deleted by the *Member*. *Open Humans* also allows *Members* to delete their entire account at any time, resulting in an immediate removal from the database, cessation of data processing activities, and permanent deletion following the automated turnover of back-up storage.

Data input and management

Data is uploaded into a *Member*'s account, which allows any joined *Projects* with requisite permissions to access this data. To be fully available to all of the possible projects that can be run on *Open Humans*, all data are stored in files that can be downloaded by users and *Projects* that have gotten permission. For any file that a *Project* deposits into a *Member*'s account, the uploading *Project* needs to specify at least a description and tags as meta data for the files.

Members can always review and access the *Data* stored in their own accounts. By default, the *Data* uploaded into their accounts is not shared with any projects but the one that deposited the data, unless and until other *Projects* are joined and specifically authorized to access this data. In addition to being able to share data with other *Projects*, *Members* can also opt-in into making the data of individual data sources publicly available on a project-by-project basis. *Data* that has been publicly shared is then discoverable through the *Open Humans* Public Data API, and is visible on a *Member*'s user profile.

Open Humans in Practice

Using this design, the platform now features a number of projects that import data directly into *Open Humans*. Among data sources that can be imported and connected are *23andMe*, *AncestryDNA*, *Fitbit*, *Runkeeper*, *Withings*, *uBiome* and a generic VCF importer for genetic data like whole exome or genome sequences. Furthermore, as a special category, the *Data Selfie* project allows *Members* to add additional data files that are not supported by a specialized project yet.

The community around the *Open Humans* platform has expanded the support to additional *Data* sources by writing their own data importers and data connections. These include a bridge to *openSNP*, and importers for data from *FamilyTreeDNA*, *Apple HealthKit*, *Gencove*, *Twitter* and the *Nightscout* (open source diabetes) community. Across these data importers, the platform supports data sources covering genetic and activity tracking data as well as recorded GPS tracks, data from glucose monitors, and social media.

The platform has grown significantly since its launch in 2015: As of May 30th 2019, 6,976 members have signed up with *Open Humans*. Of these, 2,945 members have loaded 19,949 data sets into their accounts. In cases where external data sources support the import of historical data (e.g. Fitbit, Twitter), data sets can include data that reaches back before the

launch of Open Humans. Furthermore, overall there are now 30 projects that are actively running on Open Humans, with an additional 12 projects that have already finished data collection and thus have been concluded (see Table 1 for the most heavily engaged projects).

Use Cases

To demonstrate the range of projects made possible through the platform and how the community improves the ecosystem that is growing around *Open Humans*, here we highlight some of the ongoing projects, covering participant-led research, academic research, and projects originating in the self-quantification community.

OpenAPS and Nightscout Data & Data Commons

There are a variety of open source diabetes tools and applications that have been created to aid individuals with type 1 diabetes in managing and visualizing their diabetes data from disparate devices. One such tool is Nightscout, which allows users to access continuous glucose monitoring (CGM) data. Another such example is OpenAPS, the Open Source Artificial Pancreas System, which is designed to automatically adjust an insulin pump's insulin delivery to keep users' blood glucose in a safe range overnight and between meals [73]. These tools enable real-time and retrospective data analysis of rich and complex diabetes data sets from the real world.

Traditionally, gathering this level of diabetes data would be time-consuming, expensive, and otherwise burdensome to the traditional researcher, and often pose a prohibitive barrier to researchers interested in getting started in the area of diabetes research and development. Using Open Humans, individuals from the diabetes community have created a data uploader tool called Nightscout Data Transfer Tool to enable individuals to share their CGM and related data with the Nightscout and/or OpenAPS Data Commons [74]. Sharing is done pseudonymously via random identifiers, enabling an individual to protect their privacy. Furthermore, sharing is facilitated as a single data upload may be used in multiple studies and projects. These two patient-led data commons have requirements for use that allow both traditional *or* citizen science (e.g. patient) researchers to use this data for research. These data commons were created with the goal of facilitating more access to diabetes data such as CGM datasets that are traditionally expensive to access. By doing so, they enable more researchers to explore innovations for people with diabetes. Additionally, OpenAPS is the first open source artificial pancreas system with hundreds of users, who are hoping such data sharing will facilitate better tools and better innovations for academic and commercial innovations in this space. To date, dozens of researchers and many community members have accessed and utilized data from each of these commons. Some publications and presentations have also showcased the work and the data donated by members of the community, further allowing other researchers to build on this body of work and these data sets [75] (https://openaps.org/outcomes/).

In addition to facilitating easier access to more and richer diabetes data, the Nightscout and OpenAPS communities have also been developing a series of open source tools to enable individuals to more easily work with the datasets (https://github.com/danamlewis/OpenHumansDataTools).

Linking across communities: openSNP

openSNP is a database for personal genomics data that takes a different approach than *Open Humans*. While *Open Humans* focuses on granular control in terms of whom Members share their data with, *openSNP* focuses on maximizing re-use of data, by exclusively allowing individuals to donate the raw DTC genetic test data into the public domain [71]. With already over 4,500 genetic data sets, *openSNP* is one of the largest openly crowdsourced genome databases. In addition to the genetic data, members of *openSNP* annotate their data with additional trait data. There is no integration of further data sources into *openSNP*.

Despite the differences between *openSNP* and *Open Humans*, there is overlap of members that use both platforms, with *openSNP* members having additional non-genetic public data sets in *Open Humans*. By linking the public data sets across both platforms, both ecosystems can be enriched and members can avoid having to upload their data twice.

The connection of accounts is performed by each platform providing links to the same member on the other platform: The *openSNP* project for *Open Humans* asks members for permission to read their *Open Humans* username during the authentication phase. By recording a members *Open Humans* username, it becomes possible to link the public data sets on *Open Humans* to a given *openSNP member*. Furthermore, *openSNP* deposits a link to the public *openSNP* data sets in their *Open Humans* member account. So far over 250 people have taken advantage of linking their *openSNP* and *Open Humans* accounts to each other.

Genetic Data Augmentation

Most DTC genetic testing companies genotype customers using single-nucleotide polymorphism (SNP) genotyping technology, which genotypes a fraction of the total available sites in a human genome. As any two human genomes are more than 99 percent identical, these genotyped sites are carefully selected to capture human variation across global sub-populations. These sites (or genetic *variants*) can inform customers about their genetic ancestry, predict traits such as eye color, and determine susceptibility to some recessive diseases. While DTC testing may only genotype a tiny fraction of total sites available in the genome, it's offered at a fraction of the price when compared to more comprehensive genotyping methods such as exome or genome sequencing. Until recently, individuals who wanted to know their genotypes at sites not covered by DTC testing needed to purchase a significantly more expensive genotyping test.

Genome-wide genotype imputation is an increasingly popular technique that offers a no- or low-cost alternative to comprehensive genotyping methods. In short, imputation is performed by scanning the entire genome in large intervals and using high-quality genotype calls from a large reference population to statistically determine a sample's (or samples') genotype likelihoods at missing sites based on shared genotypes with the reference population. Traditionally, genotype imputation has not been readily accessible to DTC customers because it entails a complex multi-step process requiring technical expertise and computing resources. Recently, the Michigan Imputation Server launched a free to use imputation pipeline [76]. The server was designed to be user-friendly and greatly lowered the barrier to entry for everyday DTC customers to have access to imputed genotypes.

As part of the Open Humans platform, *Imputer* is a participant-created project that performs genome-wide genotype imputation on one of a *Member's* connected genetic data sources, such as *23andMe* or *AncestryDNA*. Firstly, *Imputer* must be authorized by a *Member*; once connected, the *Imputer* interface (https://openimpute.com) allows the *Member* to select which genetic data source they would like to impute and launches the imputation pipeline in one click. *Imputer* submits the imputation job to a queue on a server where the imputation is performed. Once the job has finished, the imputed genotypes are uploaded as a *.vcf* file and an email is sent to the *Member* notifying them that their data is available. *Imputer* makes it easy

Table 1. *Open Humans* projects with more than 250 members

Project name	Description	Members	Data deposited	Data access requested
23andMe Upload	Enables members to import their 23andMe data	1202	23andMe data	-
Genevieve Genome Report	Matches a member's genome against public variant data, and invites them to contribute to shared notes.	845	-	23andMe Upload, Harvard PGP, Genome/Exome Upload, Username & public data
Harvard Personal Genome Project	Enables members to import their data from the Personal Genome Project	812	Full genome sequencing data & survey data	-
Twitter Archive Analyzer	Enables members to import their Twitter archives and analyzes them	531	Twitter archives	-
Personal Data Notebooks	Enables personal data analyses with Jupyter Notebooks	524	Jupyter Notebooks	All *Data*
Keeping Pace	Seeks to study data about how we move around, to understand how seasons and local environment influence our movement patterns.	403	-	Fitbit, Jawbone, Moves, Apple HealthKit, Runkeeper
AncestryDNA Upload	Enables members to import their AncestryDNA data	438	AncestyDNA data	-
Fitbit Connection	Connect a member's Fitbit account to add data from their Fitbit activity trackers and other Fitbit devices.	404	Data from a Fitbit account	-
GenomiX Genome Exploration	A study of how people interact with their genome data using GenomiX, a visualization tool	365	-	Username & public data
Circles	A research study that aims to discover the genetic basis for a mysterious and remarkable human trait: the areola.	321	-	23andMe, AncestryDNA, Data Selfies, Harvard PGP, Genome/Exome Upload
Gencove	Your genome app - get your ancestry, microbiome, and more! Contribute your data to OpenHumans.	311	Sequencing bam files	-
openSNP	Enables members to connect their *Open Humans* and *openSNP* accounts	308	openSNP user details	Username & public data
Nightscout Data Transfer	A tool to easily enable the upload of data from individual Nightscout databases	293	Nightscout data	-

Data was collected on 2019-04-25

for *Members* to augment their existing genetic data sources using techniques that were previously difficult to access. The *Imputer* imputation pipeline was built using genipe [77] and uses the 1000 Genomes Project [78] genotype data as the reference population.

Re-use of Public Data for Understanding Health Behavior

A research team at the Universities of Copenhagen and Geneva, the Quality of Life (QoL) Technologies Lab, has been able to perform preliminary research using public data in Open Humans. As physical inactivity is one of the strongest risk factors for preventable chronic conditions [79], the QoL Lab's goal is

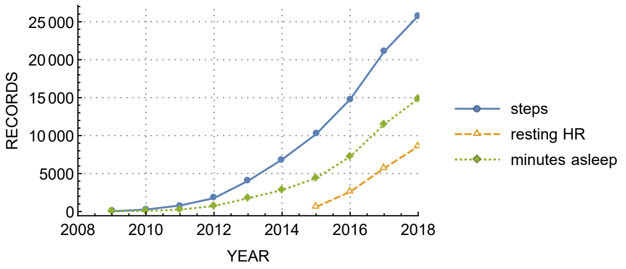

Figure 2. Self-quantification data from *Fitbit* project. Number of public records from January 2009 until October 2018 (cumulative total).

to leverage self-quantification data to assess and subsequently enhance the well-being of individuals and possibly, in the long-term, reduce the prevalence of some chronic diseases. At this stage, the QoL Lab has used the *Open Humans* public datasets of *Fitbit* and *Apple HealthKit* projects.

In *Open Humans*, individuals who donate public data uploaded from Fitbit and Apple HealthKit projects share with others the daily summaries taken with their *Fitbit* and *Apple* devices such as steps, resting heart rate (HR) and minutes asleep. The public datasets contain time series data from at least 30 members, each of whom decides whether to provide access to the aforementioned measurements. The number of records for each variable available in the *Open Humans* database varies since not all the devices record the same variables and participants may choose not to share a particular measurement (see Fig. 2).

The QoL Technologies Lab team reports that access to public data has facilitated its research planning. While the number of public datasets is smaller in terms of the number of members who give this kind of access, they are very useful for running observational studies over long periods of time and can be used to prepare data cleaning and processing methods, which can then be applied to follow-up studies. As running studies on Open Humans and accessing private data as part of a research institution requires approval from an Institutional Review Board – a potentially lengthy process – the availability of the public data allows development and testing of methods during an earlier stage of the research process. A study is now being developed based on this preliminary work. Additionally, the QoL Lab has been granted ethics approval from University of Copenhagen in November 2018 (#504-0034/18-5000).

Data re-use in genetic data visualization research

With the increasing number of individuals engaging with their genetic data, including via direct-to-consumer products, there is a need for research into how individuals interact with this data to explore and understand it. The *Human-Computer Interaction for Personal Genomics* (PGHCI) project at Wellesley College and New York University has focused on exploring these questions. Research was initially conducted by creating visualizations of genetic data interpretations based on public genetic data sets and associated reports. The research initially recruited participants via Amazon Mechanical Turk to evaluate a set of visualizations; this approach, however, was not based on participants' *own* information, which is preferred to improve experimental validity.

Open Humans provided an opportunity to work with individuals and their data in a manner that leveraged pre-existing genetic data for re-use in new research while minimizing privacy risks. A project, *GenomiX Genome Exploration*, was created in Open Humans that invited *Members* who had publicly shared their genetic data in Open Humans to engage with a custom visualization derived from their existing public data and associated interpretations. The study found various design implications in genome data engagement, including the value of affording users the flexibility to examine the same report using multiple views [80].

Personal Data Exploration

Open Humans aggregates data from multiple sources connected to individual *Members*. This makes it a natural starting point for a *Member* to explore their personal data. To facilitate this, *Open Humans* includes the *Personal Data Notebooks* project.

Through a *JupyterHub* setup (https://jupyterhub.readthedocs.io) that authenticates *Members* through their *Open Humans* accounts, *Members* can write *Jupyter Notebooks* [81] that get full access to their personal data in their web browser. This allows *Members* to explore and analyze their own data without the need to download or install specialized analysis software on their own computers. Furthermore, it allows *Members* to easily analyze data across the various data sources, for example combining data about their social media usage as well as activity tracking data from wearable devices. This allows *Members* to explore potential correlations such as whether a decrease in physical activity correlates with more time spent on social media.

As the notebooks themselves do not store any of the personal data, but rather the generic methods to access the data, they can be easily shared between *Open Humans Members* without leaking a *Member*'s personal data. This property facilitates not only the sharing of analysis methods, but also reproducible *n=1* experiments in the spirit of self-quantification.

To make these notebooks not only interoperable and reusable, but also findable and accessible [82], the sister project to the *Personal Data Notebooks* – the *Personal Data Exploratory* – was started. *Members* can upload notebooks right from their Personal Data Notebook interface to *Open Humans* and can publish them on the *Personal Data Exploratory* site with just a few clicks. The *Exploratory* publicly displays the published notebooks to the wider community and categorizes them according to the data sources used, tags and its content.

The categorization allows other *Members* to easily discover notebooks of interest. Notebooks written by other *Members* can be launched and run on a *Member*'s own personal data through the *Personal Data Notebooks*, requiring only a single click of a button. Through the close interplay between the *Personal Data Notebooks* and the shared notebook library of the *Personal Data Exploratory*, *Open Humans* offers an integrated personal data analysis environment that allows personal data to be disseminated in a private and secure way, while simultaneously growing a library of data exploration tools that can be reused by other *Members*, as shown in Figure 3.

Google search history analyzer and community review

The *Google Search History Analyzer* is a project that highlights the *Open Humans* community review process for *Projects*, demonstrating how this process can help improve not only a project that is reviewed, but also the infrastructure of *Open Humans*. The *Google Search History Analyzer* invites individuals to upload their Google Search History data, and analyze them in a quantitative way, through *Personal Data Notebooks*. Examples of analyses that users can do through the *Personal Data Notebooks* include retrieving graphs of their most common search terms and their daily or weekly evolution, as well as visualizing connections among their top search terms and their co-occurrence. One goal of this project is to raise awareness on the breadth and deeply personalized content that web searches might carry. Another long-term goal is to provide social scientists who are currently using web search history data for predicting social trends, e.g. unemployment [83], or interest in medical conditions [84], with the means to have access to a pool of individuals who can provide informed consent to the use of their search history data along with additional meta-data (e.g. de-

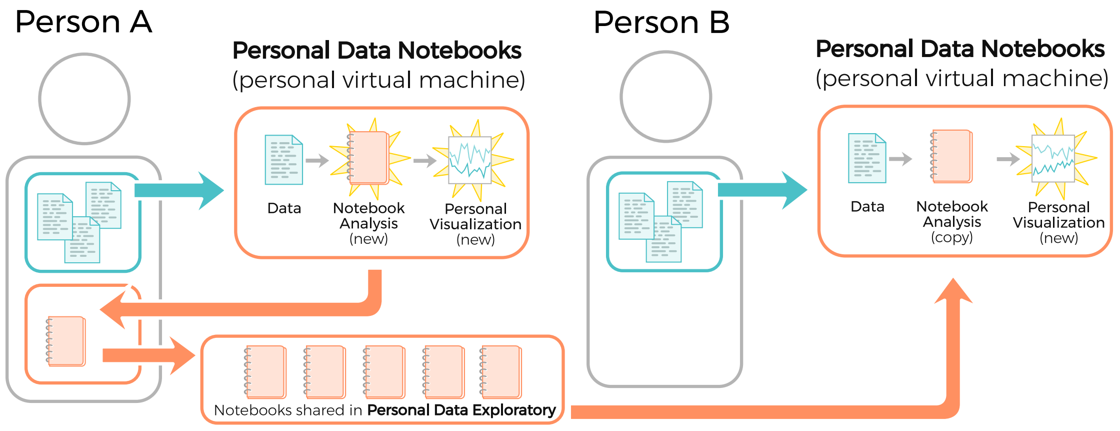

Figure 3. Personal Data Notebooks in Open Humans. Any *Member* (e.g. "Person A", left), can create a *Notebook* to explore their personal *Data* using the *Personal Data Notebooks* project. They can then choose to share a *Notebook* via the *Personal Data Exploratory*. This allows another *Member* (e.g. "Person B", right) to load a copy of the *Notebook* and run it, privately, to produce their own analysis.

mographic information, or survey questions), that could render their Google search history terms more informative.

Open Humans requires *Projects* have an "approval" to become visible and broadly available to *Members*. Prior to *Google Search History Analyzer*, this approval process was informal and internal; however, the sensitive data handled by this *Project* raised concerns regarding a need for a more formal and transparent review process – as web search history terms might carry highly personalized information, like personal interests, medical history, places a person visits, or even predictors for severe psychiatric conditions [85]. As a result, a community review process was developed for *Project* approvals going forward: new projects are shared with the larger community for public comments, inviting feedback from all *Members*. Project owners can reply to the feedback and improve their project accordingly, as well as seek help from other *Open Humans Members*. The community review continues until concerns, if any, have been resolved; no formal timeline for finding consensus exists, instead the process is adaptive to the levels of concern raised by the community members. If and when project approval occurs, this status is implemented by the administrators of the Open Humans platform. Project approval status can be re-considered at any time by opening a new review process, which may be done by any community *Member*.

As a result of this process, the *Google Search History analyzer* project was improved with added, documentation, increased clarity, and additional security implementations on the project side. Furthermore, it led to the implementation of a new feature on the *Open Humans* platform itself, enabling a project-specific override to prevent public data sharing by *Members* for this data – as requested by community review – thereby reducing the risk that these sensitive datasets might be publicly released by the *Members* accidentally.

Discussion

Participatory/community science (also known as citizen science) is a growing field that engages people in the scientific process. But while participatory science keeps growing quickly in the environmental sciences and astronomy, its development in the humanities, social sciences, and in medical research lags behind [86], despite expectations that it will make inroads into those fields [60, 87]. Both barriers in accessing personal data that is stored in commercial entities as well as legitimate ethical concerns that surround the use of personal data contribute to this slower adoption in realms that rely on access to personal information [34, 36]. *Open Humans* was designed to address many of these issues—we discuss some of them in subsequent sections.

Granular and Specific Consent

One often suggested way to mitigate the ethical concerns around the sharing of personal data in a research framework is by giving participants granular consent options [37]. In a medical context, most patients prefer to have granular control over which medical data to share and for which purposes [88, 89], especially in the context of electronic medical records [90]. Furthermore, the GDPR requires that organizations handling personal data give the individual granular consent options for how their data is used [91].

Open Humans strongly limits the platforms use of member data to an opt-in model, implementing a form of granular consent for data sharing and data use through the use of projects that *Members* can opt into. On a technical level, project organizers need to select the data sources they would like to access, and *Members* can give specific consent for that project's activities. From the perspective of *Open Humans* this produces a format for granular consent regarding the data it manages, as each potential use of data in the platform is mediated by a specific project.

Additionally, projects on *Open Humans* need to adhere to the community guidelines. In addition to mandating clarity and specificity in consent, these guidelines require projects to inform prospective participants about the level of data access they would request, how the data would be used, and what privacy and security precautions they have in place. Authorization may be withdrawn at any time, at which point projects may no longer access de-authorized data. Furthermore, projects may receive notification of erasure requests made by participants who withdraw, should they opt to support these.

Data portability

Much of health data is still stored in data silos managed by national institutions, sometimes further sub-categorized by diseases [92]. On an individual level, the situation is not much better: While medical data is usually stored in electronic records, much of a person's data is now held by the companies that run social media platforms, develop smartphone apps, or purvey

wearable devices [93]. This fragmentation—especially when coupled with a lack of data export methods—prevents individuals from authorizing new uses of their data.

Personal information management systems (PIMS) could help individuals in re-collecting and integrating their personal data from different sources [94]. The right to data portability, as encapsulated in Article 20 of the GDPR, has the potential to boost the adoption of such systems, as it guarantees individuals in the European Union a right to export the personal data they have provided to data holders in electronic and other useful formats. While Article 20 does not cover derived data, such as genetic information generated from biological samples [95], other personal data that is provided directly and thus subject to Article 20 can be highly valuable for individuals and research purposes. Additionally, Article 15 of the GDPR provides individuals with further rights to access and copies to such derived data, though without specific provisions for the format of such data. Both traditional medical research [96] as well as citizen science [97] have the potential to benefit from these data. By design, *Open Humans* works similar to a PIMS, as it allows individuals to bundle and collect their personal data from external sources. Like other PIMS, *Open Humans* is likely to benefit from any increase in data export, e.g. due to the GDPR.

While the availability of data export functions is a necessary condition for making PIMS work, it alone is not sufficient. PIMS need to support the data import on their end, either by supporting the file types or by offering support for the APIs of the external services. As file formats and APIs are not static, but can change over time, especially among popular services [98], a significant amount of effort is needed to keep data import functions into PIMS up to date. This cost keeps accumulating as the number of supported data imports keeps increasing. The modular, project-based nature of *Open Humans* allows the distribution of the workload of keeping integrations up to date, as data importers can be provided by any third party. Existing data imports on *Open Humans* already demonstrate this capability: Both the *Nightscout* and the *Apple HealthKit* data importers are examples of this. In the case of *Nightscout*, members of the diabetes community themselves built and maintain the data import into *Open Humans* to power their own data commons that overlays the *Open Humans* data storage. And the *HealthKit* import application was written by an individual *Open Humans Member* who wanted to add support for adding their own data.

Enabling individual-centric research and citizen science

Open Humans provides several benefits for citizen science efforts and individual researchers who do not work in academia. The *OpenAPS* and *Nightscout Data Commons* highlighted in the results are prime examples of how Open Humans can enable such participant-led research.

To enable research done by non-traditional researchers, the project creation workflow of Open Humans includes information for project leaders about informed consent and other key considerations. It encourages project administrators to be clear about both data management and security in a thorough community guide `https://www.openhumans.org/community-guidelines/#project`. This guide includes best practice guidelines for data security as well as details on how to communicate to participants which data access is being requested and why. It emphasizes plain language and clarity.

To further the community's sense of ownership in the *Open Humans* platform, participants are involved in the governance of the ecosystem. On a high level the community gets to elect a third of the members of the Open Humans Foundation board of directors, enabling them to exert direct influence on the larger direction of the platform.

Furthermore, *Members* of Open Humans are invited to participate in the approval of new projects that want to be shared on the platform via a community review process, as illustrated by the *Google Search History* project use case described above. This community review process parallels efforts made elsewhere to pursue participant-centred alternatives to institutional review boards [99], which at present include extremely limited input from community members. Indeed, traditional policies for project approval from an ethical standpoint have been repeatedly questioned [100], and even more so for the case of participant-centric research [101], due to inconsistent levels of engagement from non-academic members [102] and lack of participant protection and autonomy [101]. Notably the review process as implemented on *Open Humans* is less structured than traditional approaches, as it is performed by community members who choose to participate; self-selection for engagement may help maximize efficiency in a heterogeneous ecosystem. We hope this alternative design helps inform other projects seeking increased participant input in project review and oversight.

Summary

Open Humans is an active online platform for personal data aggregation and data sharing that enables citizen science and traditional academic science alike. By leaving data-sharing decisions to individual members, the platform offers a way of doing personal data-based research in an iterative, ethically sensitive way and enables individuals to engage in science as both investigators and participants.

Methods

The primary Open Humans web application, as well as data source *Projects* maintained directly by *Open Humans*, are written in Python 3 using the Django web framework. API endpoints, JSON and HTML data serialization, and OAuth2 authorization are managed by the *Django REST Framework* and *Django OAuth Toolkit* libraries. Web apps are deployed on *Heroku* and use *Amazon S3* for file storage. The *Personal Data Notebooks* JupyterHub project is deployed via *Google Cloud Platform*.

Two Python packages have been developed and distributed in the *Python Package Index* to facilitate interactions with our API: (1) *open-humans-api* provides Python functions for API endpoints, as well as command line tools for performing many standard API operations, (2) *django-open-humans* provides a reusable Django module for using *Open Humans* OAuth2 and API features.

Open Humans complies with GDPR and provides a live records of processing activities report at: https://www.openhumans.org/data-processing-activities/

Availability of source code and requirements

- Project name: Open Humans
- Project home page: `http://www.openhumans.org`
- Operating system(s): Platform independent
- Programming language: Python3
- Other requirements: full list on GitHub `https://github.com/openhumans/open-humans/`
- License: MIT

- Project name: Open Humans API
- Project home page: `https://open-humans-api.readthedocs.io/en/latest/`

- Operating system(s): Platform independent
- Programming language: Python3
- Other requirements: full list on GitHub https://github.com/openhumans/open-humans-api
- License: MIT

- Project name: Django Open Humans
- Project home page: https://github.com/OpenHumans/django-open-humans
- Operating system(s): Platform independent
- Programming language: Python3
- Other requirements: full list on GitHub
- License: MIT

Declarations

List of abbreviations

API: application programming interface CGM: Continuous Glucose Monitor DTC: Direct to Consumer GDPR General Data Protection Regulation IRB: Institutional Review Board PIMS: Personal information management systems QoL: Quality of Life

Ethical Approval

Not applicable

Consent for publication

Not applicable

Competing Interests

BGT is supported by a fellowship from *Open Humans Foundation*, which operates *Open Humans*. MPB is funded for full time work at *Open Humans Foundation* as Executive Director and President. MA is a paid consultant to Genetic Alliance and Variant Bio.

Funding

The development and operation of Open Humans has been supported through grants from the Robert Wood Johnson Foundation, John S. and James L. Knight Foundation, and Shuttleworth Foundation.

Author's Contributions

BGT: Conceptualization, Data curation, Investigation, Methodology, Project administration, Software, Supervision, Writing – original draft, Writing – review & editing MA: Supervision, Writing – review & editing KA: Data curation, Software, Validation, Writing – original draft, Writing – review & editing MD: Software, Writing – review & editing VE: Data curation, Formal analysis, Investigation, Validation, Visualization, Writing – original draft, Writing – review & editing BG: Data curation, Resources, Software, Validation TH: Methodology, Resources, Software DL: Data curation, Formal analysis, Validation, Writing – original draft, Writing – review & editing OS: Investigation, Validation, Writing – review & editing ON: Investigation, Validation AT: Data curation, Software, Validation, Writing – original draft, Writing – review & editing JB: Conceptualization, Funding acquisition, Resources, Investigation, Project administration, Supervision MPB: Conceptualization, Data curation, Funding acquisition, Investigation, Methodology, Project administration, Resources, Software, Supervision, Writing – original draft, Writing – review & editing

Acknowledgements

The authors would like to thank all members of the Open Humans community for their diverse contributions to Open Humans: Developing the process as well as platforms that link to Open Humans, sharing their personal data, advancing public knowledge sources, being active community members.

In this spirit, this manuscript was written as a community project done by and with Open Humans members following an open call for contributions.

In particular, the authors would like to thank Rosy Gupta, Manaswini Das, Jasmine Tamak and Tarannum Khan. They made valuable contributions as summer interns with Open Humans through the Outreachy internship program. The authors are grateful to Mike Escalante, who contributed in software development as well as mentoring for Outreachy.

The authors also would like to thank the reviewers – their input significantly improved the manuscript.

References

1. McCormick TH, Lee H, Cesare N, Shojaie A, Spiro ES. Using Twitter for Demographic and Social Science Research: Tools for Data Collection and Processing. Sociological Methods & Research 2015 oct;46(3):390–421. https://doi.org/10.1177/0049124115605339.
2. Özdemir V, Dove ES, Gürsoy UK, Şardaş S, Yıldırım A, Yılmaz ŞG, et al. Personalized medicine beyond genomics: alternative futures in big data—proteomics, environtome and the social proteome. Journal of Neural Transmission 2015 dec;124(1):25–32. https://doi.org/10.1007/s00702-015-1489-y.
3. Athey S. Beyond prediction: Using big data for policy problems. Science 2017 feb;355(6324):483–485. https://doi.org/10.1126/science.aal4321.
4. Cappella JN. Vectors into the Future of Mass and Interpersonal Communication Research: Big Data, Social Media, and Computational Social Science. Human Communication Research 2017 jun;43(4):545–558. https://doi.org/10.1111/hcre.12114.
5. Collins FS, Varmus H. A New Initiative on Precision Medicine. New England Journal of Medicine 2015 feb;372(9):793–795. https://doi.org/10.1056/nejmp1500523.
6. Chhibber A, Kroetz DL, Tantisira KG, McGeachie M, Cheng C, Plenge R, et al. Genomic architecture of pharmacological efficacy and adverse events. Pharmacogenomics 2014 dec;15(16):2025–2048. https://doi.org/10.2217/pgs.14.144.
7. Kummar S, Williams PM, Lih CJ, Polley EC, Chen AP, Rubinstein LV, et al. Application of Molecular Profiling in Clinical Trials for Advanced Metastatic Cancers. JNCI Journal of the National Cancer Institute 2015 feb;107(4):djv003–djv003. https://doi.org/10.1093/jnci/djv003.
8. Dilsizian SE, Siegel EL. Artificial Intelligence in Medicine and Cardiac Imaging: Harnessing Big Data and Advanced Computing to Provide Personalized Medical Diagnosis and Treatment. Current Cardiology Reports 2013 dec;16(1). https://doi.org/10.1007/s11886-013-0441-8.
9. Moon H, Ahn H, Kodell RL, Baek S, Lin CJ, Chen JJ. Ensemble methods for classification of patients for personalized medicine with high-dimensional data. Artificial

Intelligence in Medicine 2007 nov;41(3):197–207. https://doi.org/10.1016/j.artmed.2007.07.003.
10. Kohane IS. Ten things we have to do to achieve precision medicine. Science 2015 jul;349(6243):37–38. https://doi.org/10.1126/science.aab1328.
11. Wetterstrand LA, DNA Sequencing Costs: Data; 2018. https://www.genome.gov/sequencingcostsdata/.
12. Swan M. Emerging Patient-Driven Health Care Models: An Examination of Health Social Networks, Consumer Personalized Medicine and Quantified Self-Tracking. International Journal of Environmental Research and Public Health 2009 feb;6(2):492–525. https://doi.org/10.3390/ijerph6020492.
13. Gay V, Leijdekkers P. Bringing Health and Fitness Data Together for Connected Health Care: Mobile Apps as Enablers of Interoperability. Journal of Medical Internet Research 2015 nov;17(11):e260. https://doi.org/10.2196/jmir.5094.
14. Corpas M, Valdivia-Granda W, Torres N, Greshake B, Coletta A, Knaus A, et al. Crowdsourced direct-to-consumer genomic analysis of a family quartet. BMC Genomics 2015 nov;16(1). https://doi.org/10.1186/s12864-015-1973-7.
15. Regalado A, 2017 was the year consumer DNA testing blew up; 2018. https://www.technologyreview.com/s/610233/2017-was-the-year-consumer-dna-testing-blew-up/.
16. Khan R, Mittelman D. Consumer genomics will change your life, whether you get tested or not. Genome Biology 2018 aug;19(1). https://doi.org/10.1186/s13059-018-1506-1.
17. Regalado A, More than 26 million people have taken an at-home ancestry test. MIT Technology Review; 2019. https://www.technologyreview.com/s/612880/more-than-26-million-people-have-taken-an-at-home-ancestry-test/.
18. EMC, The digital universe: Driving data growth in healthcare; 2014. https://web.archive.org/web/20180525094214/https://www.emc.com/analyst-report/digital-universe-healthcare-vertical-report-ar.pdf.
19. Rozenblum R, Bates DW. Patient-centred healthcare, social media and the internet: the perfect storm? BMJ Quality & Safety 2013 feb;22(3):183–186. https://doi.org/10.1136/bmjqs-2012-001744.
20. DeAngelis S, Patient Monitoring, Big Data, and the Future of Healthcare; 2014. https://www.wired.com/insights/2014/08/patient-monitoring-big-data-future-healthcare/.
21. Weber GM, Mandl KD, Kohane IS. Finding the Missing Link for Big Biomedical Data. JAMA 2014 may;https://doi.org/10.1001/jama.2014.4228.
22. Carbon S, Champieux R, McMurry J, Winfree L, Wyatt LR, Haendel M. A Measure of Open Data: A Metric and Analysis of Reusable Data Practices in Biomedical Data Resources 2018 mar;https://doi.org/10.1101/282830.
23. Blasimme A, Fadda M, Schneider M, Vayena E. Data Sharing For Precision Medicine: Policy Lessons And Future Directions. Health Affairs 2018 may;37(5):702–709. https://doi.org/10.1377/hlthaff.2017.1558.
24. Kossmann D, Brand A, Hafen E. Health Data Cooperatives – Citizen Empowerment. Methods of Information in Medicine 2014;53(02):82–86. https://doi.org/10.3414/me13-02-0051.
25. Tenopir C, Allard S, Douglass K, Aydinoglu AU, Wu L, Read E, et al. Data Sharing by Scientists: Practices and Perceptions. PLoS ONE 2011 jun;6(6):e21101. https://doi.org/10.1371/journal.pone.0021101.
26. Simpson C, Goldenberg A, Culverhouse R, Daley D, Igo R, Jarvik G, et al. Practical Barriers and Ethical Challenges in Genetic Data Sharing. International Journal of Environmental Research and Public Health 2014 aug;11(8):8383–8398. https://doi.org/10.3390/ijerph110808383.
27. Lye CT, Forman HP, Gao R, Daniel JG, Hsiao AL, Mann MK, et al. Assessment of US Hospital Compliance With Regulations for Patients' Requests for Medical Records. JAMA Network Open 2018 oct;1(6):e183014. https://doi.org/10.1001/jamanetworkopen.2018.3014.
28. Wong J, Henderson T. How portable is portable? Exercising the GDPR's Right to Data Portability. In: Proceedings of the 2018 ACM International Joint Conference and 2018 International Symposium on Pervasive and Ubiquitous Computing and Wearable Computers United States: ACM; 2018. p. 911–920.
29. Blumenthal D, Tavenner M. The "Meaningful Use" Regulation for Electronic Health Records. New England Journal of Medicine 2010 aug;363(6):501–504. https://doi.org/10.1056/nejmp1006114.
30. Hert PD, Papakonstantinou V, Malgieri G, Beslay L, Sanchez I. The right to data portability in the GDPR: Towards user-centric interoperability of digital services. Computer Law & Security Review 2018 apr;34(2):193–203. https://doi.org/10.1016/j.clsr.2017.10.003.
31. Recommendation on Return of Individual Research Results; 2016. https://www.hhs.gov/ohrp/sachrp-committee/recommendations/attachment-b-return-individual-research-results/index.html.
32. Wong CA, Hernandez AF, Califf RM. Return of Research Results to Study Participants. JAMA 2018 aug;320(5):435. https://doi.org/10.1001/jama.2018.7898.
33. Mason PH. The Ethics of Biomedical Big Data. Journal of Bioethical Inquiry 2017 oct;14(4):571–574. https://doi.org/10.1007/s11673-017-9812-y.
34. Ross MW, Iguchi MY, Panicker S. Ethical aspects of data sharing and research participant protections. American Psychologist 2018 feb;73(2):138–145. https://doi.org/10.1037/amp0000240.
35. Haeusermann T, Greshake B, Blasimme A, Irdam D, Richards M, Vayena E. Open sharing of genomic data: Who does it and why? PLOS ONE 2017 may;12(5):e0177158. https://doi.org/10.1371/journal.pone.0177158.
36. Wang S, Jiang X, Singh S, Marmor R, Bonomi L, Fox D, et al. Genome privacy: challenges, technical approaches to mitigate risk, and ethical considerations in the United States. Annals of the New York Academy of Sciences 2016 sep;1387(1):73–83. https://doi.org/10.1111/nyas.13259.
37. Evans BJ. Power to the People: Data Citizens in the Age of Precision Medicine. Vanderbilt J Entertain Technol Law 2017;19(2):243–265.
38. Hart K, A new data scandal: How ancestry DNA firms share your most intimate secrets. Axios; 2019. https://www.axios.com/dna-test-results-privacy-genetic-data-sharing-4687b1a0-f527-425c-ac html.
39. Might M, Might CC. What happens when N = 1 and you want plus 1? Prenatal Diagnosis 2016 dec;37(1):70–72. https://doi.org/10.1002/pd.4975.
40. Stacchiotti S, Gronchi A, Fossati P, Akiyama T, Alapetite C, Baumann M, et al. Best practices for the management of local-regional recurrent chordoma: a position paper by the Chordoma Global Consensus Group. Annals of Oncology 2017 feb;28(6):1230–1242. https://doi.org/10.1093/annonc/mdx054.
41. PEER is Transforming Health Systems. Genetic Alliance, Inc.; 2015. https://www.peerplatform.org/idea/.
42. Samerski S. Individuals on alert: digital epidemiology and the individualization of surveillance. Life Sciences, Society and Policy 2018 jun;14(1). https://doi.org/10.1186/s40504-018-0076-z.

43. Cox J, 70,000 OkCupid Users Just Had Their Data Published; 2016. http://motherboard.vice.com/read/70000-okcupid-users-just-had-their-data-published.
44. Zimmer M. “But the data is already public”: on the ethics of research in Facebook. Ethics and Information Technology 2010 jun;12(4):313–325. https://doi.org/10.1007/s10676-010-9227-5.
45. Zook M, Barocas S, danah boyd, Crawford K, Keller E, Gangadharan SP, et al. Ten simple rules for responsible big data research. PLOS Computational Biology 2017 mar;13(3):e1005399. https://doi.org/10.1371/journal.pcbi.1005399.
46. Jouhki J, Lauk E, Penttinen M, Sormanen N, Uskali T. Facebook’s Emotional Contagion Experiment as a Challenge to Research Ethics. Media and Communication 2016 oct;4(4):75. https://doi.org/10.17645/mac.v4i4.579.
47. Hunter D, Evans N. Facebook emotional contagion experiment controversy. Research Ethics 2016 jan;12(1):2–3. https://doi.org/10.1177/1747016115626341.
48. Flick C. Informed consent and the Facebook emotional manipulation study. Research Ethics 2015 aug;12(1):14–28. https://doi.org/10.1177/1747016115599568.
49. Bruns A, Facebook Shuts the Gate after the Horse Has Bolted, and Hurts Real Research in the Process; 2018. https://medium.com/@Snurb/facebook-research-data-18662cf2cacb.
50. Golder S, Ahmed S, Norman G, Booth A. Attitudes Toward the Ethics of Research Using Social Media: A Systematic Review. Journal of Medical Internet Research 2017 jun;19(6):e195. https://doi.org/10.2196/jmir.7082.
51. Pocock MJO, Tweddle JC, Savage J, Robinson LD, Roy HE. The diversity and evolution of ecological and environmental citizen science. PLOS ONE 2017 apr;12(4):e0172579. https://doi.org/10.1371/journal.pone.0172579.
52. Vayena E, Tasioulas J. “We the Scientists”: a Human Right to Citizen Science. Philosophy & Technology 2015 jun;28(3):479–485. https://doi.org/10.1007/s13347-015-0204-0.
53. McKinley DC, Miller-Rushing AJ, Ballard HL, Bonney R, Brown H, Cook-Patton SC, et al. Citizen science can improve conservation science, natural resource management, and environmental protection. Biological Conservation 2017 apr;208:15–28. https://doi.org/10.1016/j.biocon.2016.05.015.
54. Conrad CC, Hilchey KG. A review of citizen science and community-based environmental monitoring: issues and opportunities. Environmental Monitoring and Assessment 2010 jul;176(1-4):273–291. https://doi.org/10.1007/s10661-010-1582-5.
55. Zevin M, Coughlin S, Bahaadini S, Besler E, Rohani N, Allen S, et al. Gravity Spy: integrating advanced LIGO detector characterization, machine learning, and citizen science. Classical and Quantum Gravity 2017 feb;34(6):064003. https://doi.org/10.1088/1361-6382/aa5cea.
56. Haklay M. Citizen Science and Volunteered Geographic Information: Overview and Typology of Participation. In: Crowdsourcing Geographic Knowledge Springer Netherlands; 2012.p. 105–122. https://doi.org/10.1007/978-94-007-4587-2_7.
57. Dickinson H, Fortson L, Lintott C, Scarlata C, Willett K, Bamford S, et al. Galaxy Zoo: Morphological Classification of Galaxy Images from the Illustris Simulation. The Astrophysical Journal 2018 feb;853(2):194. https://doi.org/10.3847/1538-4357/aaa250.
58. Khatib F, Cooper S, Tyka MD, Xu K, Makedon I, Popovic Z, et al. Algorithm discovery by protein folding game players. Proceedings of the National Academy of Sciences 2011 nov;108(47):18949–18953. https://doi.org/10.1073/pnas.1115898108.
59. Ranard BL, Ha YP, Meisel ZF, Asch DA, Hill SS, Becker LB, et al. Crowdsourcing—Harnessing the Masses to Advance Health and Medicine, a Systematic Review. Journal of General Internal Medicine 2013 jul;29(1):187–203. https://doi.org/10.1007/s11606-013-2536-8.
60. Rowbotham S, McKinnon M, Leach J, Lamberts R, Hawe P. Does citizen science have the capacity to transform population health science? Critical Public Health 2017 nov;p. 1–11. https://doi.org/10.1080/09581596.2017.1395393.
61. Mader LB, Harris T, Kläger S, Wilkinson IB, Hiemstra TF. Inverting the patient involvement paradigm: defining patient led research. Research Involvement and Engagement 2018 jul;4(1). https://doi.org/10.1186/s40900-018-0104-4.
62. Vayena E, Brownsword R, Edwards SJ, Greshake B, Kahn JP, Ladher N, et al. Research led by participants: a new social contract for a new kind of research. Journal of Medical Ethics 2015 mar;42(4):216–219. https://doi.org/10.1136/medethics-2015-102663.
63. Katapally TR, Bhawra J, Leatherdale ST, Ferguson L, Longo J, Rainham D, et al. The SMART Study, a Mobile Health and Citizen Science Methodological Platform for Active Living Surveillance, Integrated Knowledge Translation, and Policy Interventions: Longitudinal Study. JMIR Public Health and Surveillance 2018 mar;4(1):e31. https://doi.org/10.2196/publichealth.8953.
64. Wicks P, Vaughan TE, Massagli MP, Heywood J. Accelerated clinical discovery using self-reported patient data collected online and a patient-matching algorithm. Nature Biotechnology 2011 apr;29(5):411–414. https://doi.org/10.1038/nbt.1837.
65. McDonald D, Hyde E, Debelius JW, Morton JT, Gonzalez A, Ackermann G, et al. American Gut: an Open Platform for Citizen Science Microbiome Research. mSystems 2018;3(3). https://msystems.asm.org/content/3/3/e00031-18.
66. McGowan ML, Choudhury S, Juengst ET, Lambrix M, Settersten RA, Fishman JR. “Let’s pull these technologies out of the ivory tower”: The politics, ethos, and ironies of participant-driven genomic research. BioSocieties 2017 mar;12(4):494–519. https://doi.org/10.1057/s41292-017-0043-6.
67. Levitan B, Getz K, Eisenstein EL, Goldberg M, Harker M, Hesterlee S, et al. Assessing the Financial Value of Patient Engagement. Therapeutic Innovation & Regulatory Science 2017 jul;52(2):220–229. https://doi.org/10.1177/2168479017716715.
68. Swan M. The Quantified Self: Fundamental Disruption in Big Data Science and Biological Discovery. Big Data 2013 jun;1(2):85–99. https://doi.org/10.1089/big.2012.0002.
69. Swan M. Health 2050: The Realization of Personalized Medicine through Crowdsourcing, the Quantified Self, and the Participatory Biocitizen. Journal of Personalized Medicine 2012 sep;2(3):93–118. https://doi.org/10.3390/jpm2030093.
70. Yuan J, Gordon A, Speyer D, Aufrichtig R, Zielinski D, Pickrell J, et al. DNA.Land is a framework to collect genomes and phenomes in the era of abundant genetic information. Nature Genetics 2018 jan;50(2):160–165. https://doi.org/10.1038/s41588-017-0021-8.
71. Greshake B, Bayer PE, Rausch H, Reda J. openSNP–A Crowdsourced Web Resource for Personal Genomics. PLoS ONE 2014 mar;9(3):e89204. https://doi.org/10.1371/journal.pone.0089204.
72. Ball MP, Thakuria JV, Zaranek AW, Clegg T, Rosenbaum AM, Wu X, et al. A public resource facilitating clinical

use of genomes. Proceedings of the National Academy of Sciences 2012 jul;109(30):11920–11927. https://doi.org/10.1073/pnas.1201904109.

73. Lewis D, and SL. Real-World Use of Open Source Artificial Pancreas Systems. Journal of Diabetes Science and Technology 2016 aug;10(6):1411–1411. https://doi.org/10.1177/1932296816665635.
74. Lewis DM, Ball MP. OpenAPS Data Commons on Open Humans 2017 9;https://figshare.com/articles/OpenAPS_Data_Commons_on_Open_Humans/5428498.
75. Lewis DM, Leibrand S, Street TJ, Phatak SS. Detecting Insulin Sensitivity Changes for Individuals with Type 1 Diabetes. Diabetes 2018 may;67(Supplement 1):79–LB. https://doi.org/10.2337/db18-79-lb.
76. Das S, Forer L, Schönherr S, Sidore C, Locke AE, Kwong A, et al. Next-generation genotype imputation service and methods. Nature Genetics 2016 Aug;48:1284 EP –. http://dx.doi.org/10.1038/ng.3656.
77. Lemieux Perreault LP, Legault MA, Asselin G, Dubé MP. genipe: an automated genome-wide imputation pipeline with automatic reporting and statistical tools. Bioinformatics 2016;32(23):3661–3663. http://dx.doi.org/10.1093/bioinformatics/btw487.
78. Consortium TGP, Auton A, Abecasis GR, Altshuler (Co-Chair) DM, Durbin (Co-Chair) RM, Bentley DR, et al. A global reference for human genetic variation. Nature 2015 Sep;526:68 EP –. http://dx.doi.org/10.1038/nature15393, article.
79. WH D, CE D, RC B. Chronic disease prevention: Tobacco avoidance, physical activity, and nutrition for a healthy start. JAMA 2016;316(16):1645–1646. +http://dx.doi.org/10.1001/jama.2016.14370.
80. Westendorf L, Shaer O, Pollalis C, Verish C, Nov O, Ball MP. Exploring Genetic Data Across Individuals: Design and Evaluation of a Novel Comparative Report Tool. Journal of Medical Internet Research 2018 sep;20(9):e10297. https://doi.org/10.2196/10297.
81. Kluyver T, Ragan-Kelley B, Pérez F, Granger B, Bussonnier M, Frederic J, et al. Jupyter Notebooks – a publishing format for reproducible computational workflows. In: Loizides F, Schmidt B, editors. Positioning and Power in Academic Publishing: Players, Agents and Agendas IOS Press; 2016. p. 87 – 90.
82. Wilkinson MD, Dumontier M, Aalbersberg IJ, Appleton G, Axton M, Baak A, et al. The FAIR Guiding Principles for scientific data management and stewardship. Scientific Data 2016 mar;3:160018. https://doi.org/10.1038/sdata.2016.18.
83. D'Amuri F, Marcucci J. The predictive power of Google searches in forecasting US unemployment. International Journal of Forecasting 2017 oct;33(4):801–816. https://doi.org/10.1016/j.ijforecast.2017.03.004.
84. Brigo F, Trinka E. Google search behavior for status epilepticus. Epilepsy & Behavior 2015 aug;49:146–149. https://doi.org/10.1016/j.yebeh.2015.02.029.
85. III JFG, Lester D. Using google searches on the internet to monitor suicidal behavior. Journal of Affective Disorders 2013 jun;148(2-3):411–412. https://doi.org/10.1016/j.jad.2012.11.004.
86. Kullenberg C, Kasperowski D. What Is Citizen Science? – A Scientometric Meta-Analysis. PLOS ONE 2016 jan;11(1):e0147152. https://doi.org/10.1371/journal.pone.0147152.
87. Power to the Patients: Co-design of Community-based Research; 2018. http://blogs.plos.org/blog/2018/08/09/power-to-the-patients-co-design-of-community-based-research/.
88. Schwartz PH, Caine K, Alpert SA, Meslin EM, Carroll AE, Tierney WM. Patient Preferences in Controlling Access to Their Electronic Health Records: a Prospective Cohort Study in Primary Care. Journal of General Internal Medicine 2014 dec;30(S1):25–30. https://doi.org/10.1007/s11606-014-3054-z.
89. Grando MA, Murcko A, Mahankali S, Saks M, Zent M, Chern D, et al. A Study to Elicit Behavioral Health Patients' and Providers' Opinions on Health Records Consent. The Journal of Law, Medicine & Ethics 2017 jun;45(2):238–259. https://doi.org/10.1177/1073110517720653.
90. Caine K, Hanania R. Patients want granular privacy control over health information in electronic medical records. Journal of the American Medical Informatics Association 2013 jan;20(1):7–15. https://doi.org/10.1136/amiajnl-2012-001023.
91. Nati M, Mayer S, Capossele A, Missier P. Toward Trusted Open Data and Services. Internet Technology Letters 2018 jul;p. e69. https://doi.org/10.1002/itl2.69.
92. The Global Alliance for Genomics and Health. A federated ecosystem for sharing genomic, clinical data. Science 2016 jun;352(6291):1278–1280. https://doi.org/10.1126/science.aaf6162.
93. Althoff T. Population-Scale Pervasive Health. IEEE Pervasive Computing 2017 oct;16(4):75–79. https://doi.org/10.1109/mprv.2017.3971134.
94. Allard T, Bouadi T, Duguépéroux J, Sans V. From Self-data to Self-preferences: Towards Preference Elicitation in Personal Information Management Systems. In: Personal Analytics and Privacy. An Individual and Collective Perspective Springer International Publishing; 2017.p. 10–16. https://doi.org/10.1007/978-3-319-71970-2_2.
95. Taylor MJ, Wallace SE, Prictor M. United Kingdom: transfers of genomic data to third countries. Human Genetics 2018 aug;137(8):637–645. https://doi.org/10.1007/s00439-018-1921-0.
96. Rumbold JMM, Pierscionek B. The Effect of the General Data Protection Regulation on Medical Research. Journal of Medical Internet Research 2017 feb;19(2):e47. https://doi.org/10.2196/jmir.7108.
97. Quinn P. Is the GDPR and Its Right to Data Portability a Major Enabler of Citizen Science? Global Jurist 2018 jun;18(2). https://doi.org/10.1515/gj-2018-0021.
98. Xavier L, Brito A, Hora A, Valente MT. Historical and impact analysis of API breaking changes: A large-scale study. In: 2017 IEEE 24th International Conference on Software Analysis, Evolution and Reengineering (SANER) IEEE; 2017. https://doi.org/10.1109/saner.2017.7884616.
99. Grant AD, Wolf GI, Nebeker C. Approaches to governance of participant-led research: a qualitative case study. BMJ Open 2019 apr;9(4):e025633. https://doi.org/10.1136/bmjopen-2018-025633.
100. Mhaskar R, Pathak E, Wieten S, Guterbock T, Kumar A, Djulbegovic B. Those Responsible for Approving Research Studies Have Poor Knowledge of Research Study Design: a Knowledge Assessment of Institutional Review Board Members. Acta Informatica Medica 2015;23(4):196. https://doi.org/10.5455/aim.2015.23.196-201.
101. Wilson E, Kenny A, Dickson-Swift V. Ethical challenges of community based participatory research: exploring researchers' experience. International Journal of Social Research Methodology 2017 mar;21(1):7–24. https://doi.org/10.1080/13645579.2017.1296714.
102. Klitzman R. Institutional Review Board Community Members. Academic Medicine 2012 jul;87(7):975–981. https://doi.org/10.1097/acm.0b013e3182578b54.
