# Supplementary material for: Open Humans: A platform for participant-centered research and personal data exploration
Source: Gigascience. 2019 Jun 26;8(6):giz076. doi: 10.1093/gigascience/giz076 (PMC6593360; doi:10.1093/gigascience/giz076)
Supplement: giz076_Response_to_Reviewer_Comments_Original_Submission [file giz076_response_to_reviewer_comments_original_submission.pdf]

Author's Response To Reviewer Comments

Close

Responses to reviewers

We thank the reviewers for offering us their extremely helpful comments on the earlier version of this manuscript. They were central in shaping the current version and helped to sharpen its focus and improve it substantially. A point-by-point response to the comments can be found below:

Reponses to Reviewer #1

Comment: Abstract P1L36-37. I am struck by the framing of this ethical problem as the responsibility of data subjects. I assume this is intentional and would appreciate a little more, perhaps in the introduction, as to what is entailed in this responsibility?
Response: The word choice here of "responsibility" was probably not ideal, as we aren't attempting to assign this to any particular group - we've updated to frame this as balancing risk/benefit, which we believe Open Humans helps achieve through its inclusion of the community in various aspects of its operation.

Comment: Abstract P1L42-43. I am not sure if the framing of the ethical problem is resolved by the description of the utility of Open Humans. While overall, I suggest deepening the ethical problems presented, another alternative is to leave it out all together.
Response: Based on feedback from our other reviewer, we have taken the route of "deepening" this, specifically by articulating specific features and how these related to rights protected by the EU GDPR.

Comment: P2C2L6-9. It would help me if parties were more clearly stated. I think you mean researchers not research and it isn't clear to me that commercial data sources have interests but rather the companies that hold these resources do, right?
Response: We updated the manuscript to improve the language in response, thank you!

Comment: P2C2 Participant Involvement. It is unclear to me what the purpose of this section is.
Response: Some text in this section has been added and updated to connect this section to the potential benefits to research that can come from participant insights.

Comment: P2C1 Data Silos. Most of the descriptive language is written in the passive voice which I understand may be the norm but in my opinion, it unintentionally highlights how interests and responsibilities are dissociated or dis-located from stakeholders. For instance, in the section on Data Silos, it remains unclear for whom Data Silos are a problem and whose interests have created and maintained these silos. Again, this sort of analysis might help identify or locate solutions rather than only set up a problem that Open Human's solves. My point here is that the developers of Open Humans need not rely on a somewhat limited ethical analysis to justify its existence and argue for its utility.
Response: We've expanded this to touch on other reasons that may drive silos, including technical challenges and costs in data management, and incentives for restricting access.

Comment: P2C1L44-49. While I agree this is accurate reflection of the scope of literature, the issues raised by "big data" research now extend far beyond the common risks relayed in a consent process.
Reponse: We agree that there is a need for more than individual consent; we've added discussion of how a community review process in open humans enables the community as a whole make decisions about acceptable projects, which arguably extends individual consent models to add collective governance.

Comment: P2C1L49-51. I agree that this is an important issue but this single statement citing Barbara Evans sounds a little like a strawman. My sense is that through the efforts of many patient-driven organizations, patient and participant-driven research has increased a great deal in the past decade or so. Perhaps this ought to be recognized especially given that many of the authors have been critical to the development of this movement. Also, the next section on participant involvement seems at odds

with the argument so some clarification might help readers understand the nuances.
Response: We've added references to recognize more of the current state of the patient/participant-driven research to make the section overall more clear.

Comment: P2C2L53-61. While I totally agree and appreciate these key points to the participant-centered approach to research, in all honesty, I did not come to these conclusions based on the above exposition. I suggest moving this up as the scaffold for the introduction and reorganize based on these points.
Reponse: We've rewritten and expanded portions of the introduction to better connect the various reasons supporting a participant-centered approach to research and how these are achieved with Open Humans

Comment: P3C1L30-36. These are the main points I think readers need in the introduction to help us understand the need for Open Humans. I suggest you spend more time explaining these points and characterizing the evidence of these important assertions.
Response: We have expanded the introduction to address these points earlier in the text, see also the comments above.

Comment: P3C2L46-50. Could you explain the rationale behind this feature and briefly describe if more detailed information is conveyed about the IRB approval or review/determination?
Response: We expanded this section to explain the rational and describe how the information is conveyed to members

Comment: P4C2L25-27. This is an important statement, at least to me, but it would be helpful to reiterate how privacy is maintained, I'm assuming because its pseudonymous?
Response: We have expanded this point, making it clearer how privacy is maintained.

Comment: P4C2L27-30. Again, what are the simple requirements?
Response: We don't really know: the requirements are managed by the patient-led commons, who helped contribute to this manuscript text. We've edited it to more accurately describe the scope of potential data use in their commons.

Comment: P5C1L58-C2L59. So what are the ethical implications of this use case? I think an important point to highlight is that privacy may be a nominal issue with members of efforts like Open Humans as they often have a greater than average interest in research benefits than maintaining individual privacy. Further, I'm under the impression that personal privacy is less of a concern for many or rather our sense of what is private is changing. Assuming I'm understanding the argument, what I'm confused about is that the ethical analysis presented in the background assumes that privacy is of central perhaps even sole concern. Also, there are many other ethical issues that open humans both addresses possibly in a positive way and potentially raises as risks to members and even society. So, I would welcome that analysis alongside this nice introduction to the platform or I would not rest the argument for the platform on a relatively narrow ethical frame.
Response: We have rephrased this section to make clear the differences between openSNP & Open Humans. Open Humans focuses on giving participants the choice to keep their data private and give them control with whom to share their data. As part of enabling choice it also enables public sharing of data (e.g. see the work the QoL labs presents in the results) and supporting linking further public data from openSNP Is part of this.

Comment: P6C2L16-21. Do you mean the public data are being used as training sets for the algorithms? Are there any risks of bias based on these sorts of uses?
Response: We have reworded this section to clarify that the public data is not mainly used for training of machine learning algorithms or making research conclusions, but for the design of further data collection and processing tools that will be used in later studies. E.g. anticipate file formats, data artifacts, and such

Comment; P6C1L44-45. So are there any ethical issues related to the application of OAuth2 to these particular use cases or overall? This isn't a trick question, I have no idea but would encourage the authors to consider based on their expertise.
Response: We've explained what "OAuth2" means, hopefully providing a better explanation of this and other aspects of "projects" in general.

Comment:P7C2L9-11. Agreed, but does it also make it harder for bad actors to use these data? It would be great if the authors could help us think about this potential trade off.

Response: The wording here has been improved a bit to articulate individual authorization for new data uses. There is still a potential for data aggregations being performed outside the authorization of the individual, despite apparent silos. This can and does happen without individual involvement in various places, but it's probably too much to address here.

Comment: P7C1 Discussion. I would like the authors to consider the following in the discussion and possibly the introduction. (1) Given that most people who engage in citizen science in the biomedical research space are likely to subscribe to the value of openness and sharing of samples, data, tools, etc., I wonder if focusing on privacy as key ethical barrier is on target and sufficient. For instance, many of the challenges to genomic research articulated by historically vulnerable populations have to do with offensive data uses, lack of control, lack of direct benefit, differential benefit based on SES, risks to groups, etc. Again, a critical analysis of how this resource might increase or decrease such risks involved in citizen science would contribute to the larger project of extending citizen science or patient-led research to community-led research. Of course, I understand this might been outside the bounds of this manuscript but that preclude some consideration. (2) Ivery much appreciate Open Humans as a tool that addresses the practical problem of bridging/linking/aggregating. I have no problems with this argument yet I wonder if it is somewhat naive to assume that bridging as a practical benefit does not also risk other ethical challenges. For example, the ease of bridging to pre-selected resources blurs the line between simply linking resources and advancing particular interpretations of the data, in fact, one's own data. If I understand Open Humans, it is a tool that automates protocols for linking and sharing intended to facilitate citizen science and patient-led research. The practical benefits are clear. But what are the risks associated with more automated linking and sharing?
Response: Regarding (1) we've clarified that the platform goes beyond merely addressing privacy concerns by also enabling the co-creation of research and community review processes regarding projects on the site -- hopefully these expansions are relevant here. (2) While there is substantial automation, Open Humans can also be considered a "high friction" environment from the perspective of consent, as opt-in decisions are required for each new project. New risk associated with Open Humans may have less to do with automation, but rather in the decentralization and democratization of projects: by enabling patient-led projects, it may be possible we enable projects that are "riskier" than traditional academic research. That said, this is speculative; indeed, a converse claim might also arguably be true -- that community led projects are more likely to benefit communities. Because this is all speculative, we haven't expanded the paper with these thoughts.

Comment: P7C2 Enabling individual-centric research and citizen science. This section is very helpful and references a number of mechanisms that begin to address, at least on an individual level, issues such as "to what uses", "control", "governance", etc. I would love to either see this description expanded and moved up into the initial description of the resource (maybe before or around P2C2L57) and or these functional benefits better incorporated and explicated in the use cases.
Response: We have articulated more specifically which features exist in terms of individual control for data sharing as well as community governance. A new use case that highlights the governance mechanics has been added as well

Comment: P8C1L13-16. It is unclear to me how it is "an ethical way" especially as it isn't clear to me what an "unethical way" would entail. I think some pieces are presented but this argument could be much stronger and clearer. I get that the benefits are assumed here to some extent, I've been in the same place when engaging in resource development, but perhaps a greater consideration of potential benefits and harms might help balance the focus on privacy and individual control. Generally when we conduct ethical analysis we consider autonomy (where privacy sits), risks (as potential harms as well as increasingly benefits), and justice. Notably. others might argue for other principles and values. While such a comprehensive analysis isn't the focus of this manuscript, incorporating the insights of such an analysis would, in my opinion, strengthen the argument for Open Humans and signal/evidence robust consideration by its designers and authors.
Response: That is a fair point, the language has been toned down accordingly

REVIEWER #2

Comment: WHAT CONSTITUTES CONTROL? Firstly, under the General Data Protection Regulation, the individual has the following rights: right to be informed, right of access, right to rectification, right to be forgotten, right to restriction of processing, right to data portability, the right to object and, albeit less relevant in this context, rights in relation to automated decision-making. Yet, in relation to scientific research, most Member States of the European Union allow for the right of access, the right to rectification, and the right to restriction of processing to be denied. The article very briefly mentions

data access, within the context of human subjects research, to be recommended but not legally required. However, it does not make mention of the other two deniable rights (right to rectification + right to restrict processing).
Response: We've added information about the ability to withdraw from projects, which results in an immediate cessation of data access available to that project (without deleting that data in their Open Humans account), as well as a notification of data erasure requests (if supported by the project). We've also clarified that data deletion is available, but optional. Taken together, these support GDPR rights of restriction of processing and erasure -- although these are are limited to what we can accomplish on our end, and must be mediated by what the projects themselves support. The act of performing rectification is dependent on what data source projects do, as Open Humans is acting as a generic receiver of various data that is uploaded by projects.

Comment: It leads to the first main question: what exactly constitutes control? How does Open Humans define control? The article mentions and describes a granular consent and privacy model. However, consent is important, but merely a legal basis for processing. How does Open Humans guarantee the other individual rights as granted by the General Data Protection Regulation? The right to information is shortly described on page 7, and so is the right of data portability, but, if full control is the desirable route, it means guaranteeing all rights granted. However, in the context of reproducibility of scientific research, granting all rights does not seem feasible. In particular, the right of rectification and the right to restrict processing seem problematic.
Response: We've tried to reduce the vague language about "control" here, adding more functional details regarding data sharing management by the individual, as well as our support for data erasure notifications made to projects upon the withdrawal of authorizations.

Comment: GRANULAR CONSENT IS DIFFERENT FROM SPECIFIC CONSENT. The GDPR requires consent to be freely given, specific, informed and unambiguous (see article 7 and recital 32). Granular consent is needed when one service is involved with multiple processing operations for multiple purposes. In such a case, consent is required for every purpose of processing. This is referred to as granular consent. Whilst closely related, granular consent is therefore different from specific consent.
Response: That's fair, we didn't mean to imply that research projects within Open Humans have granular consent. Rather, our intent is to describe Open Humans itself as supporting granularity in consent through the ongoing management of various specific consents made for each project. We've updated the text to clarify this distinction.

Comment: RIGHT TO DATA PORTABILITY IS LIMITED TO DATA PROVIDED BY THE INDIVIDUAL. The right to data portability is regarded to have the potential to boost the adoption of a system where individuals can recollect and integrate their personal data from different sources, 'as it guarantees individuals in the European Union a right to export their personal data in electronic and other useful formats'.
Response: This is a good point. Article 20 limits the right to portability to data provided by data subjects and e.g. genetic data derived from biological samples is excluded from this. We clarified this point and also point out that the rights to data access and copies of the derived data as given by Article 15 can be useful for getting access to personal data as well.

Close
