# Supplementary material for: Open Humans: A platform for participant-centered research and personal data exploration
Source: Gigascience. 2019 Jun 26;8(6):giz076. doi: 10.1093/gigascience/giz076 (PMC6593360; doi:10.1093/gigascience/giz076)
Supplement: giz076_Response_to_Reviewer_Comments_Revision_1 [file giz076_response_to_reviewer_comments_revision_1.pdf]

Author's Response To Reviewer Comments

Close

We have addressed the additional comments of the reviewers.

The LaTeX source with tracked changes for the manuscript can be viewed at
https://www.overleaf.com/read/kybdrqszgpzs

For the individual comments:

1) P3C2P2. Under Results...Second sentence could be improved. Try this "Platform members import data about themselves from various sources into their Open Humans account. They can then explore their aggregated data and share it with citizen scientists and academic researchers."
This is a great idea, we have incorporated this change to improve the sentence.

2) P4C2P2. Is that on a project by project basis or global across all projects the member has joined?
This is on a project-by-project basis and we have rephrased this to make it clearer.

3) P9C1P2. The sentence "While Article 20 does not..." needs to be cleaned up.
Very good point, we have cleaned it up.

4) P9C1P4. participant-led or -lead?
This should have been participant-led and we have fixed it.

We also want to thank the reviewers for all the time they have put into reviewing our manuscript and the extremely useful feedback they have given. We feel that the manuscript has improved dramatically thanks to their input. We would like to ask the reviewers whether they would like to be listed with their names in the acknowledgement? Given all their work, we would like to fully credit them for their work if they agree.

Close
