# Supplementary material for: Open Humans: A platform for participant-centered research and personal data exploration
Source: Gigascience. 2019 Jun 26;8(6):giz076. doi: 10.1093/gigascience/giz076 (PMC6593360; doi:10.1093/gigascience/giz076)
Supplement: giz076_Reviewer_1_Report_Original_Submission — Joon-Ho Yu -- 11/28/2018 Reviewed [file giz076_reviewer_1_report_original_submission.pdf]

Reviewer Report

Title: Open Humans: A platform for participant-centered research and personal data exploration

Version: Original Submission **Date:** 11/28/2018

Reviewer name: Joon-Ho Yu

Reviewer Comments to Author:

Thank you for the opportunity to review this manuscript. Overall, I appreciate this argument for and description of Open Humans. Broadly, the manuscript would benefit from greater attention to writing and organization. As my comments describe below, the "ethical analysis" offered is narrowly focused and appears to serve as a justification for the resource; yet, in its current state, I think the ethical analysis either should be removed or expanded. Ideally, the manuscript would be strengthened by a deepening and broadening of ethical considerations.
Note that I use P(page)C(column)L(lines) to locate my comments for the authors.
1. Abstract P1L36-37. I am struck by the framing of this ethical problem as the responsibility of data subjects. I assume this is intentional and would appreciate a little more, perhaps in the introduction, as to what is entailed in this responsibility?
2. Abstract P1L42-43. I am not sure if the framing of the ethical problem is resolved by the description of the utility of Open Humans. While overall, I suggest deepening the ethical problems presented, another alternative is to leave it out all together.
3. P2C2L6-9. It would help me if parties were more clearly stated. I think you mean researchers not research and it isn't clear to me that commercial data sources have interests but rather the companies that hold these resources do, right?
4. P2C2 Participant Involvement. It is unclear to me what the purpose of this section is.
5. P2C1 Data Silos. Most of the descriptive language is written in the passive voice which I understand may be the norm but in my opinion, it unintentionally highlights how interests and responsibilities are dissociated or dis-located from stakeholders. For instance, in the section on Data Silos, it remains unclear for whom Data Silos are a problem and whose interests have created and maintained these silos. Again, this sort of analysis might help identify or locate solutions rather than only set up a problem that Open Human's solves. My point here is that the developers of Open Humans need not rely on a somewhat limited ethical analysis to justify its existence and argue for its utility.
6. P2C1L44-49. While I agree this is accurate reflection of the scope of literature, the issues raised by "big data" research now extend far beyond the common risks relayed in a consent process.
7. P2C1L49-51. I agree that this is an important issue but this single statement citing Barbara Evans sounds a little like a strawman. My sense is that through the efforts of many patient-driven organizations, patient and participant-driven research has increased a great deal in the past decade or so. Perhaps this ought to be recognized especially given that many of the authors have been critical to the development of this movement. Also, the next section on participant involvement seems at odds with the argument so some clarification might help readers understand the nuances.
8. P2C2L53-61. While I totally agree and appreciate these key points to the participant-centered

approach to research, in all honesty, I did not come to these conclusions based on the above exposition. I suggest moving this up as the scaffold for the introduction and reorganize based on these points.

9. P3C1L30-36. These are the main points I think readers need in the introduction to help us understand the need for Open Humans. I suggest you spend more time explaining these points and characterizing the evidence of these important assertions.
10. P3C2L46-50. Could you explain the rationale behind this feature and briefly describe if more detailed information is conveyed about the IRB approval or review/determination?
11. P4C2L25-27. This is an important statement, at least to me, but it would be helpful to reiterate how privacy is maintained, I'm assuming because its pseudonymous?
12. P4C2L27-30. Again, what are the simple requirements?
13. P5C1L58-C2L59. So what are the ethical implications of this use case? I think an important point to highlight is that privacy may be a nominal issue with members of efforts like Open Humans as they often have a greater than average interest in research benefits than maintaining individual privacy. Further, I'm under the impression that personal privacy is less of a concern for many or rather our sense of what is private is changing. Assuming I'm understanding the argument, what I'm confused about is that the ethical analysis presented in the background assumes that privacy is of central perhaps even sole concern. Also, there are many other ethical issues that open humans both addresses possibly in a positive way and potentially raises as risks to members and even society. So, I would welcome that analysis alongside this nice introduction to the platform or I would not rest the argument for the platform on a relatively narrow ethical frame.
14. P6C2L16-21. Do you mean the public data are being used as training sets for the algorithms? Are there any risks of bias based on these sorts of uses?
15. P6C1L44-45. So are there any ethical issues related to the application of OAuth2 to these particular use cases or overall? This isn't a trick question, I have no idea but would encourage the authors to consider based on their expertise.
16. P7C2L9-11. Agreed, but does it also make it harder for bad actors to use these data? It would be great if the authors could help us think about this potential trade off.
17. P7C1 Discussion. I would like the authors to consider the following in the discussion and possibly the introduction. (1) Given that most people who engage in citizen science in the biomedical research space are likely to subscribe to the value of openness and sharing of samples, data, tools, etc., I wonder if focusing on privacy as key ethical barrier is on target and sufficient. For instance, many of the challenges to genomic research articulated by historically vulnerable populations have to do with offensive data uses, lack of control, lack of direct benefit, differential benefit based on SES, risks to groups, etc. Again, a critical analysis of how this resource might increase or decrease such risks involved in citizen science would contribute to the larger project of extending citizen science or patient-led research to community-led research. Of course, I understand this might been outside the bounds of this manuscript but that preclude some consideration. (2) I very much appreciate Open Humans as a tool that addresses the practical problem of bridging/linking/aggregating. I have no problems with this argument yet I wonder if it is somewhat naive to assume that bridging as a practical benefit does not also risk other ethical challenges. For example, the ease of bridging to pre-selected resources blurs the line between simply linking resources and advancing particular

interpretations of the data, in fact, one's own data. If I understand Open Humans, it is a tool that automates protocols for linking and sharing intended to facilitate citizen science and patient-led research. The practical benefits are clear. But what are the risks associated with more automated linking and sharing?
18. P7C2 Enabling individual-centric research and citizen science. This section is very helpful and references a number of mechanisms that begin to address, at least on an individual level, issues such as "to what uses", "control", "governance", etc. I would love to either see this description expanded and moved up into the initial description of the resource (maybe before or around P2C2L57) and or these functional benefits better incorporated and explicated in the use cases.
19. P8C1L13-16. It is unclear to me how it is "an ethical way" especially as it isn't clear to me what an "unethical way" would entail.   I think some pieces are presented but this argument could be much stronger and clearer. I get that the benefits are assumed here to some extent, I've been in the same place when engaging in resource development, but perhaps a greater consideration of potential benefits and harms might help balance the focus on privacy and individual control. Generally when we conduct ethical analysis we consider autonomy (where privacy sits), risks (as potential harms as well as increasingly benefits), and justice. Notably. others might argue for other principles and values. While such a comprehensive analysis isn't the focus of this manuscript, incorporating the insights of such an analysis would, in my opinion, strengthen the argument for Open Humans and signal/evidence robust consideration by its designers and authors.

Methods

Are the methods appropriate to the aims of the study, are they well described, and are necessary controls included? Choose an item.

Conclusions

Are the conclusions adequately supported by the data shown? Choose an item.

Reporting Standards

Does the manuscript adhere to the journal's guidelines on minimum standards of reporting? Choose an item.

Choose an item.

Statistics

Are you able to assess all statistics in the manuscript, including the appropriateness of statistical tests used? Choose an item.

Quality of Written English

Please indicate the quality of language in the manuscript: Choose an item.

Declaration of Competing Interests

Please complete a declaration of competing interests, considering the following questions:

- Have you in the past five years received reimbursements, fees, funding, or salary from an organisation that may in any way gain or lose financially from the publication of this manuscript, either now or in the future?
- Do you hold any stocks or shares in an organisation that may in any way gain or lose financially from the publication of this manuscript, either now or in the future?
- Do you hold or are you currently applying for any patents relating to the content of the manuscript?
- Have you received reimbursements, fees, funding, or salary from an organization that holds or has applied for patents relating to the content of the manuscript?
- Do you have any other financial competing interests?
- Do you have any non-financial competing interests in relation to this paper?

If you can answer no to all of the above, write 'I declare that I have no competing interests' below. If your reply is yes to any, please give details below.

I declare that I have no competing interests.

I agree to the open peer review policy of the journal. I understand that my name will be included on my report to the authors and, if the manuscript is accepted for publication, my named report including any attachments I upload will be posted on the website along with the authors' responses. I agree for my report to be made available under an Open Access Creative Commons CC-BY license (http://creativecommons.org/licenses/by/4.0/). I understand that any comments which I do not wish to be included in my named report can be included as confidential comments to the editors, which will not be published.

I agree to the open peer review policy of the journal

To further support our reviewers, we have joined with Publons, where you can gain additional credit to further highlight your hard work (see: https://publons.com/journal/530/gigascience). On publication of this paper, your review will be automatically added to Publons, you can then choose whether or not to claim your Publons credit. I understand this statement.

Yes Choose an item.
