# Supplementary material for: Open Humans: A platform for participant-centered research and personal data exploration
Source: Gigascience. 2019 Jun 26;8(6):giz076. doi: 10.1093/gigascience/giz076 (PMC6593360; doi:10.1093/gigascience/giz076)
Supplement: giz076_Reviewer_2_Report_Original_Submission — Birgit Wouters -- 12/11/2018 Reviewed [file giz076_reviewer_2_report_original_submission.pdf]

Reviewer Report

Title: Open Humans: A platform for participant-centered research and personal data exploration

Version: Original Submission **Date:** 12/11/2018

Reviewer name: Birgit Wouters

Reviewer Comments to Author:

In this paper, the authors have presented an innovative solution to the complex and multi-faceted problem of sharing personal (health) data. Open Humans, a community-based platform, serves multiple aims:
(1) to be ethically justifiable:
a. by focusing upon granular, individual consent for each single project, thereby avoiding the issue of compatible purposes for secondary/tertiary/… processing;
b. by putting individuals in control of their personal dataset; and
c. by involving them in the governance of the ecosystem;
(2) to enable both academic and citizen-led research; and
(3) to break open existing data silos and allow for the merging of datasets.
Serving these aims simultaneously is undoubtedly ambitious. Yet, the authors have demonstrated how Open Humans is designed to do just that. The community-based platform has clearly been carefully designed, and the presentation of the design and the use cases is clear, well-written and easy to follow. Whilst Open Humans is an interesting and promising project, my comments center around the ethical justifiability of this community-based platform. Further clarification and/or elaboration on these comments is strongly recommended.
One important goal of Open Humans is for research to be driven by the individuals the data come from by putting them into control of their data. The level of control is described as 'full control'. In addition, putting the participant into control of their data is regarded as important taking into account the more sensitive context of precision medicine. Under "Data Silos", the authors also mention that, next to other legislation, the General Data Protection Regulation is applicable and that the right of data portability has the potential to break open these silos.
My main critique is that the article takes into account insufficiently the particularities of the General Data Protection Regulation.
WHAT CONSTITUTES CONTROL?
Firstly, under the General Data Protection Regulation, the individual has the following rights: right to be informed, right of access, right to rectification, right to be forgotten, right to restriction of processing, right to data portability, the right to object and, albeit less relevant in this context, rights in relation to automated decision-making. Yet, in relation to scientific research, most Member States of the European Union allow for the right of access, the right to rectification, and the right to restriction of processing to be denied.
The article very briefly mentions data access, within the context of human subjects research, to be recommended but not legally required. However, it does not make mention of the other two deniable

rights (right to rectification + right to restrict processing).
It leads to the first main question: what exactly constitutes control? How does Open Humans define control? The article mentions and describes a granular consent and privacy model. However, consent is important, but merely a legal basis for processing. How does Open Humans guarantee the other individual rights as granted by the General Data Protection Regulation? The right to information is shortly described on page 7, and so is the right of data portability, but, if full control is the desirable route, it means guaranteeing all rights granted. However, in the context of reproducibility of scientific research, granting all rights does not seem feasible. In particular, the right of rectification and the right to restrict processing seem problematic.
Further clarification/elaboration on this issue is required. Is full control the route Open Humans wants to take, or is Open Humans implementing a limited control for the individual? Apart from granular consent, what other forms of control does Open Humans offer?
GRANULAR CONSENT IS DIFFERENT FROM SPECIFIC CONSENT
The GDPR requires consent to be freely given, specific, informed and unambiguous (see article 7 and recital 32). Granular consent is needed when one service is involved with multiple processing operations for multiple purposes. In such a case, consent is required for every purpose of processing. This is referred to as granular consent. Whilst closely related, granular consent is therefore different from specific consent.
However, in the context of Open Humans, it is doubtful that a situation will arise where one research project will process data for more than one purpose, and thus require granular consent. Research projects work on the basis of a specific research question and/or purpose.
RIGHT TO DATA PORTABILITY IS LIMITED TO DATA PROVIDED BY THE INDIVIDUAL
The right to data portability is regarded to have the potential to boost the adoption of a system where individuals can recollect and integrate their personal data from different sources, 'as it guarantees individuals in the European Union a right to export their personal data in electronic and other useful formats'.
However, Article 20 of the GDPR limits the right to data portability to the personal data that the data subject himself/herself has provided to the controller. Data provided by the data controller do not fall under the scope of the right to data portability.
The argument that the right to data portability can lead to the breaking up from the different data silos is therefore less convincing.

Methods

Are the methods appropriate to the aims of the study, are they well described, and are necessary controls included? Choose an item.

Conclusions

Are the conclusions adequately supported by the data shown? Choose an item.

Reporting Standards

Does the manuscript adhere to the journal's guidelines on minimum standards of reporting? Choose an item.

Choose an item.

Statistics

Are you able to assess all statistics in the manuscript, including the appropriateness of statistical tests used? Choose an item.

Quality of Written English

Please indicate the quality of language in the manuscript: Choose an item.

Declaration of Competing Interests

Please complete a declaration of competing interests, considering the following questions:

- Have you in the past five years received reimbursements, fees, funding, or salary from an organisation that may in any way gain or lose financially from the publication of this manuscript, either now or in the future?
- Do you hold any stocks or shares in an organisation that may in any way gain or lose financially from the publication of this manuscript, either now or in the future?
- Do you hold or are you currently applying for any patents relating to the content of the manuscript?
- Have you received reimbursements, fees, funding, or salary from an organization that holds or has applied for patents relating to the content of the manuscript?
- Do you have any other financial competing interests?
- Do you have any non-financial competing interests in relation to this paper?

If you can answer no to all of the above, write 'I declare that I have no competing interests' below. If your reply is yes to any, please give details below.

I declare that I have no competing interests.

I agree to the open peer review policy of the journal. I understand that my name will be included on my report to the authors and, if the manuscript is accepted for publication, my named report including any attachments I upload will be posted on the website along with the authors' responses. I agree for my report to be made available under an Open Access Creative Commons CC-BY license (http://creativecommons.org/licenses/by/4.0/). I understand that any comments which I do not wish to be included in my named report can be included as confidential comments to the editors, which will not be published.

I agree to the open peer review policy of the journal

To further support our reviewers, we have joined with Publons, where you can gain additional credit to further highlight your hard work (see: https://publons.com/journal/530/gigascience). On publication of this paper, your review will be automatically added to Publons, you can then choose whether or not to claim your Publons credit. I understand this statement.

Yes Choose an item.
